# Multifunctional, TNF-α and IFN-γ-Secreting CD4 and CD8 T Cells and CD8^High^ T Cells Are Associated With the Cure of Human Visceral *Leishmania*sis

**DOI:** 10.3389/fimmu.2021.773983

**Published:** 2021-10-28

**Authors:** Lorranny Santana Rodrigues, Aline Silva Barreto, Lays Gisele Santos Bomfim, Marcos Couto Gomes, Nathalia Luisa Carlos Ferreira, Geydson Silveira da Cruz, Lucas Sousa Magalhães, Amélia Ribeiro de Jesus, Clarisa B. Palatnik-de-Sousa, Cristiane Bani Corrêa, Roque Pacheco de Almeida

**Affiliations:** ^1^ Department of Medicine, Federal University of Sergipe, Immunology Investigative Institute (III), National Insitute of Science and Technology (INCT), National Council for Scientific and Technological Development (CNPq), Aracaju, Brazil; ^2^ Graduate Program in Health Sciences, Federal University of Sergipe, Sergipe, Brazil; ^3^ Division of Immunology and Molecular Biology Laboratory, University Hospital/Brazilian Hospital Services Company (EBSERH), Federal University of Sergipe, Sergipe, Brazil; ^4^ Institute of Microbiology Paulo de Góes, Federal University of Rio de Janeiro, Immunology Investigative Institute (III), National Insitute of Science and Technology (INCT), National Council for Scientific and Technological Development (CNPq), Rio de Janeiro, Brazil; ^5^ Laboratory of Biology and Immunology of Cancer and Leishmania, Department of Morphology, Federal University of Sergipe, São Cristóvão, Brazil

**Keywords:** cellular immunity, immunophenotyping, T-cell subsets, multifunctional, human visceral leishmaniasis, *Leishmania (L.) infantum chagasi*

## Abstract

Visceral leishmaniasis (VL) is a chronic and often fatal disease caused by protozoans of the genus *Leishmania* that affects millions of people worldwide. Patients with symptomatic VL have an impaired anti-*Leishmania*-specific CD4^+^ T-cell response, which is reversed after clinical cure. In contrast, the quality of the CD4^+^ and CD8^+^ T-cell responses involved in resistance and/or cure of VL relies on the capability of these cells to activate polyfunctional and memory responses, which are associated with the simultaneous production of three cytokines: IFN-γ, IL-2, and TNF-α. Models for the development of CD4 and CD8 T-cell quality in memory and protection to leishmaniasis have been described previously. We aimed to assess the functionality of the T cells involved in the recovery of the immune suppression throughout the VL treatment. Therefore, we cultured peripheral blood mononuclear cells (PBMCs) from VL patients and healthy controls *in vitro* with soluble *Leishmania* antigen (SLA). Cell surface markers and intracellular cytokine production were determined on days 7, 14, 21, 30, 60, 90, and 180 after the beginning of chemotherapy. We observed that the frequencies of CD4^+^TNF-α^+^IFN-γ^+^ and the multifunctional CD4^+^IL-2^+^TNF-α^+^IFN-γ^+^, together with CD4^+^TNF-α^+^ and CD4^+^IFN-γ^+^ T cells, increased throughout and at the end of the treatment, respectively. In addition, enhanced frequencies of CD8^+^IL-2^+^TNF-α^+^IFN-γ^+^ and CD8^+^TNF-α^+^IFN-γ T cells were also relevant in the healing process. Noteworthy, the frequencies of the CD4^+^ and CD8 central-memory T cells, which produce IL-2, TNF-α, and IFN-γ and ensure the memory response against parasite reinfection, are significantly enhanced in cured patients. In addition, the subset of the non-functional CD8^Low^ population is predominant in VL untreated patients and decreases along the chemotherapy treatment. In contrast, a CD8^High^ subset increased towards the cure. Furthermore, the cure due to treatment with meglumine antimoniate or with liposomal amphotericin B was associated with the recovery of the T-cell immune responses. We described the evolution and participation of functional T cells during the treatment of patients with VL. Our results disclosed that the clinical improvement of patients is significantly associated with the participation of the CD4^+^ and CD8^+^ cytokine-secreting T cells.

## Introduction

Visceral leishmaniasis (VL) is a severe and frequently lethal parasite infection caused by the *Leishmania* (*L.*) *donovani*, *Leishmania* (*L.*) *infantum*, and *Leishmania* (*L.*) *infantum chagasi* complex. Between 200,000 and 400,000 new cases of VL are recorded annually worldwide (1). Of these new annual cases, 95% are concentrated in 10 countries: Bangladesh, Brazil, China, Ethiopia, India, Kenya, Nepal, Somalia, South Sudan, and Sudan (2). In the tropical regions of South America, VL is associated with the infection by *L.* (*L.*) *infantum chagasi*. In Brazil, this disease is present in almost all States, but there is a predominance of cases in the Northeast region of the country (3, 4).

VL is fatal if untreated after the onset of symptoms (5). Its main symptoms include high fever, loss of weight, hepatomegaly, splenomegaly, cachexia, anemia, leukopenia, thrombocytopenia, hypergammaglobulinemia (2, 3), and the progressive suppression of the T CD4^+^ cellular immune response, which comprises both the decrease of the total CD4 T-cell counts (6–9) and the decrease of the CD4 *Leishmania*-specific T-cell responses (10–12). The relevance of the CD4 response in this disease is so remarkable that reduced CD4 T-cell counts have become very important markers of active VL and of VL relapse (9), while the reestablishment of the normal amounts of CD4 T cells, several months after cure, is considered a marker of successful treatment (6, 11).

In addition, regarding the regulatory CD4 T-cell response, Treg cells were found to accumulate in the bone marrow of symptomatic VL patients from India. These Treg lymphocytes expand in response to *L.* (*L.*) *donovani* antigens and produce IL-10, which inhibits the T-cell response (13, 14). In agreement, the frequencies of CD4^+^CD25^High^ and CD4^+^Foxp3^+^ Treg cells were reduced in the peripheral blood mononuclear cell (PBMC) compartment of symptomatic subjects (15).

Furthermore, the relevance of the CD8 response during the disease, relapse, and cure was also studied. CD8 T cells were considered to be involved in not only protection, natural resistance, and cure (11, 16–18) but also in the immunopathogenesis of human VL (11, 16, 19). When naïve CD8 T cells become activated, they can take one of three different pathways: 1) they can secrete TNF-α and IFN-γ, 2) they can secrete cytotoxic granules, or 3) they can kill the infected cells through the Fas/FasL subsystem (20). In the *L.* (*L.*) *donovani* infection, CD8 T cells have been shown to provide resistance to the onset and to the spread of the infection though the secretion of IFN-γ, perforin, and granzyme (20). Healed patients (11, 21, 22) and asymptomatic DTH+ subjects (11) showed higher proportions of CD8 T cells. Furthermore, cured patients showed higher levels of TNF-α and IFN-γ, higher proportions of activated CD8 T cells (21), and enhanced levels of granzyme (21, 22).

Many studies have described the characteristic cytokine profile of VL patients. Immunological hallmarks of VL are the suppression of the effective Th1 immune response against *Leishmania* parasites, the presence of an elevated Th2 response associated with increased severity, and high levels of anti-inflammatory cytokines, such as IL-10 (12, 23). In contrast, the protective immune response is due to the upregulation of the Th1 response that occurs after treatment (8, 24). Anti-*Leishmania* immunity is dependent on the sustained rates of CD4 and CD8 T cells that are responsible for the secretion of cytokines involved in immunomodulation (23). Furthermore, VL patients show a characteristic suppression of the delayed type of hypersensitivity and absence of lymphocyte proliferation in response to leishmanial antigens.

Although the correlation between the cytokine profile and the clinical status of VL has been thoroughly explored, the correlation between the healing process and the differentiation of the CD4 and CD8 T-cell responses is less understood. Despite the clear definition of Th1 and Th2 responses, little is known about the functional profile of CD4^+^ T cells in VL. However, in cutaneous leishmaniasis, the quality of the Th1 response is associated with the production of cytokines by CD4^+^ T cells (25, 26). Seder’s model for the development of the CD4 Th1 response proposes that CD4 naïve T cells, after contact with the specific antigen, gradually differentiate into central-memory T (TCM) cells, based on their capacity to produce three cytokines: IL-2, TNF-α, and IFN-γ (27). Cells start this process by being single producers of IL-2 or TNF-α and are followed by double producers of TNF-α and IL-2 and, later on, by the multifunctional triple producers of IFN-γ, TNF-α, and IL-2. All of them can be considered to be TCM cells, as they are long-lasting and can respond rapidly in a second encounter with the antigen. These multifunctional T cells show a high production of IFN-γ and TNF-α, which confer on them, besides their memory functions and excellent effector and proliferation functions (26). After that, these TCM cells can further differentiate into double producers of TNF-α and IFN-γ, IFN-γ, and IL-2 and finally single producers of IFN-γ, which can be considered as terminal effector cells (TEM). The IFN-γ producer TEM cells can undergo apoptosis (25, 26). For instance, a successful vaccine against VL should induce multifunctional cells that will be able to proliferate and generate memory and effector T cells (11, 25). On the other hand, while TEM cells disappear when the infection is cured, TCM cells remain, suggesting that these cells should be the objective of vaccine development and the marker of VL cure.

On the other hand, Seder’s model for the differentiation of the CD8 T-cell response proposes that after the first contact with the antigen, the CD8 naïve T cells transform into activated effector CD8 T cells, which secrete IFN-γ and display cytotoxic activities (26). Similar to what was described for CD4 T cells, the CD8 lymphocytes submitted to continuous antigenic stimulation can further differentiate into CD8 TCM cells, which are memory T cells that secrete IL-2, TNF-α, and IFN-γ. These TCM cells might differentiate into double producers TEM cells of TNF-α and IFN-γ and, finally, into single producers of IFN-γ cells that can undergo apoptosis (26).

Clinical data regarding the differentiation of the CD4 and CD8 T-cell responses during the disease are still insufficient, but there is an indication of reduced frequencies of CD4 and CD8 activated antigen-specific T cells in symptomatic cases (15). Moreover, the frequencies of long-lasting CD4-memory T cells have been reported to be lower in symptomatic cases and higher in healed patients 10 years after being cured (15). Such an enduring memory T-cell response could be due to either the re-exposition of the individual to new infections in endemic areas or the persistence of the parasite (15). In contrast, increased frequencies of *Leishmania*-specific CD8 T cells secreting only IL-2 or IL-2 and TNF-α, and of memory CD8 T cells secreting IL-2, TNF-α, and IFN-γ, were found in symptomatic *L.* (*L.*) *infantum chagasi* infected-patients, indicating that the CD8 cytokine-secreting T cells are probably involved in the control of the early VL infection (11).

Another interesting aspect of the role of CD8 T cells during pathology not yet explored for VL is the variation of the CD8^Low^ population. This phenotype shows a low expression of the CD8 marker, low cytotoxic activity, low expression of granzyme and perforin, and a moderate expression of IFN-γ. Furthermore, CD8^Low^ has been found in increased proportions during hepatitis B infection (28), Chagas disease (29), and malaria (30) and has been related to the rapid progression of HIV infection (31).

Considering the importance of the CD4 and CD8 T-cell immune responses in the healing process of VL, we investigated the differentiation and evolution of the CD4^+^ and CD8^+^ cytokine-secreting T-cell populations during treatment and identified among them the main functional T cells, with the goal of finding new correlates of VL cure.

## Materials and Methods

### Ethical Statement

This study was approved by the Research and Ethics Committee of the Federal University of Sergipe (UFS)-University Hospital, Aracaju, Sergipe State (SE), Brazil (CAAE 0162.0.107.000-09). The experiments were performed according to the ethical standards of the Declaration of Helsinki and followed the guidelines and regulations of the Brazilian National Council of Health resolution 196/96. All participants gave their written informed consent.

### Study Population and Follow-Up

The study was performed in the Laboratory of Immunology and Molecular Biology of the UFS University Hospital, SE, Brazil, between January 2018 and December 2019. Blood samples were collected from 18 VL patients whose age ranged from 20 to 59 years (mean = 39 years). Patients were diagnosed based on the classical VL symptoms (fever, weight loss, spleen and liver enlargement, anemia, and pancytopenia). Clinical diagnosis was confirmed by serum reactivity to the rK39 antigen (KalazarDetect^®^) Rapid Test, INBIOS International Inc., Seattle, WA) and by the growth of *Leishmania* parasites in cultures of blood or bone marrow samples, using NNN media (Sigma-Aldrich) (32). After diagnosis, patients were treated daily intravenously with meglumine antimoniate (MA; 20 mg/kg/day) (Glucantime^®^ Sanofi), for 21 days, or with liposomal amphotericin B (LAMB; 3 mg/kg/day) for 5–7 days (AmBisome, Gilead). Only patients displaying more severe symptoms of VL were eligible for treatment with LAMB (32, 33). Liver and spleen sizes were evaluated through palpation by two observers and reported as the distance to the rib border in cm at diagnosis (D0) and 7 (D7), 14 (D14), 21 (D21), 30 (D30), 60 (D60), 90 (D90), and 180 (D180) days after the beginning of treatment.

Blood samples were used for hemogram and leukogram assessments and for PBMC fractionation. Peripheral blood was collected before specific *Leishmania* treatment (day 0) and on days 7, 14, 21, 30, 60, 90, and 180 after beginning the chemotherapy treatment. We also collected blood from 13 healthy controls who live in the same VL endemic area and whose ages ranged from 20 to 60 years. Control individuals had no previous history of infectious diseases for at least a month before this study and were not under treatment with immunosuppressive drugs at the time of blood collection.

### Intracellular Cytokine Staining

PBMC isolation was performed by Ficoll Paque^®^ (Sigma, USA) density gradient centrifugation on. Samples of 10^6^ PBMCs/well were cultured in Roswell Park Memorial Institute (RPMI) 1640 (Gibco^®^, USA) medium supplemented with 10% fetal calf serum and 1% of penicillin/streptomycin. Cells were stimulated with soluble *Leishmania* antigen (SLA) of *L.* (*L.*) *infantum chagasi* (10 µg/ml) or with no addition and cultured at 37°C for 6 h in a humidified atmosphere with 5% CO_2_, followed by the addition of Brefeldin at 1 µg/ml final concentration following the manufacturer’s instructions (GolgiPlug™ BD Biosciences Pharmingen, USA) and an additional 12-h incubation. After this incubation, cells were washed with phosphate-buffered saline (PBS), centrifuged at 405 g for 5 min at 4°C, and blocked with 2% fetal goat serum and 2% fetal bovine serum in PBS for 20 min (11).

The cells were treated with the following antibodies for surface staining: Anti-human CD3 PeCy7 (Clone HIT3a, BioLegend^®^, USA), Anti-human CD4 V500 (Clone RPA-T4, BD Horizon™, USA), Anti-human CD8 Pe-Cy™5 (Clone HIT8a, BD Pharmingen™, USA), Anti-human CD45 APCCy7 (Clone HI100, BioLegend^®^), and Anti-human CCR7 PE (Clone GO43H7, BioLegend^®^) for 30 min at 2°C–8°C in the dark, after which they were fixed and permeabilized with Cytofix/Cytoperm buffer (BD Biosciences Pharmingen) for 20 min. Intracellular staining was carried out by incubating the cells with Rat Anti-human IL-2 BV421 (clone MQ1-17H12, BD Horizon™), Anti-human TNF-α Alexa Fluor 647 (Clone Mab11, BioLegend^®^), and anti-IFN-γ Alexa Fluor 488 (Clone 4S.B3, BioLegend^®^), for 30 min at 4°C in the dark. Then they were washed with Perm Wash buffer (BD Biosciences Pharmingen) and PBS. Finally, the cells were centrifuged and suspended in PBS. Samples were acquired in a FACSCanto II™ flow cytometer with at least 30,000 events and analyzed using FlowJo software version 7.0 (Becton Dickinson, San Jose, CA, USA).

### Analysis Strategy of Flow Cytometry

Following the recommendations of Seder et al. (26) for multiparameter cytometry analysis, we used several hierarchical steps to identify the functional subpopulations of CD4 and CD8 T cells based on the expression of the following markers: CD3, CD4, CD8, CD45RA, CCR7, IL-2, TNF-α, and IFN-γ. First, we identified the single cells and then selected the lymphocyte cells among them (**Supplementary Figure 1**). After that, we gated the CD3-positive cells and among them identified their CD4 and CD8 subsets. Additionally, from each CD4^+^ and CD8^+^ subset, we excluded the CCR7^+^ and CD45RA^+^ cells, which are the naïve T lymphocytes. Then only the effector and memory T cells remained, and so we evaluated the production of the pro-inflammatory cytokines IL-2, TNF-α, and IFN-γ by T cells. Boolean gating was used to generate combinations of cytokine expression and types of lymphocytes in order to identify lymphocytes expressing one cytokine and any combination of two cytokines or three cytokines (**Supplementary Figure 1**).

In this study, the frequencies and the integrated mean fluorescence intensity (iMFI) of each type of cytokine-producing cells were analyzed. iMFI is a widely used parameter that is composed of the product of the frequency of a particular cytokine expressing T cell and the mean fluorescence intensity (MFI) of the same cell. Thus, this multiplication is the ideal parameter to measure the quality of cytokine production in relation to the number of producing cells.

### Statistical Analysis

The differences between the frequencies and iMFI cytokine secretion of CD4^+^ and CD8^+^ T cells of the controls and each time of treatment of all patients were compared by Mann–Whitney. Additionally, the Wilcoxon matched-pairs signed rank test was used to compare different times of treatment. We also used the IC95% for the specific comparison of total frequencies and iMFI of patients treated either with MA or LAMB. The two-way ANOVA Sidak test was used to compare the frequencies of the CD8^High^ and CD8^Low^ populations along the treatment, and Student’s *t*-test was used to compare each time of treatment with the frequencies of controls. We used GraphPad Prism 9 software, and differences were considered statistically significant for *p* < 0.05. Furthermore, we used Spearman’s two-tailed test for correlation analysis and plotted the results using the corrplot package (v0.84) running under R (v3.6.1) in RStudio (1.1.456).

## Results

### Clinical Improvements

Patients with VL were evaluated before treatment (D0) and on days 7 (D7), 14 (D14), 21 (D21), 30 (D30), 60 (D60), 90 (D90), and 180 (D180) after the beginning of the treatment. Treatments with LAMB and MA lasted until D7 and D21, respectively. All patients showed clinical improvement after treatment, as shown in **Table 1**. We do not show the complete clinical results of the 18 patients because it was not possible to collect blood for hemogram at all times, from six to seven patients who had profound anemia and cachexia. Also, nine patients did not return for control at day 180. Typical VL symptoms, such as increases in spleen and liver sizes and decreased counts of hemoglobin, hematocrit, platelets, leukocytes, neutrophils, lymphocytes, eosinophils, and monocytes, were observed in patients at D0. The chemotherapy treatment was successful and resulted in a decrease in the sizes of the spleens and livers and increases in the values of all other variables. The sizes of the spleens and livers started to decrease and were significantly different from those of untreated patients on D7. Similarly, the platelet, leukocyte, and lymphocyte counts had increased by D7. On the other hand, the hemoglobin and hematocrit levels only started to increase on D14, the monocytes increased on D21, and the neutrophils and eosinophils only increased as of D30 after beginning of the treatment. All parameters, which were monitored along the time, recovered their normal values by D180, when patients were considered cured from VL (**Table 1**). Our results indicate that the chemotherapy treatment, which lasted from 7 to 21 days, was very successful since improvements in clinical parameters were noted as early as at the end of the first week.

**Table 1:** Clinical outcomes of VL patients.

Clinical outcomes	D0 (n = 12)	D7 (n = 11)		D14 (n = 11)		D21 (n = 12)		D30 (n = 11)		D60 (n = 12)		D90 (n = 10)		D180 (n = 9)	
	Mean (SE)	Mean (SE)	*p <*	Mean (SE)	*p <*	Mean (SE)	*p <*	Mean (SE)	*p <*	Mean (SE)	*p <*	Mean (SE)	*p <*	Mean (SE)	*p <*
**Spleen (cm)**	4.8 (1.38)	3.6 (1.2)	0.015	2.2 (1.1)	0.031	2.4 (1.0)	0.015	1.6 (0.8)	0.007	0.45 (0.45)	0.015	0.45 (0.45)	0.046	0 (0)	0.031
**Liver (cm)**	3.7 (0.6)	2.2 (0.5)	0.001	1.6 (0.7)	0.007	2.6 (1.0)	0.085	2.9 (0.9)	0.031	2.3 (1.0)	0.066	0.27 (0.27)	0.003	0.1 (0.1)	0.003
**Hemoglobin (g/dl)**	8.7 (0.38)	8.7 (0.47)	0.764	10 (0.49)	0.046	10.2 (0.55)	0.004	11.5 (0.52)	0.001	13.5 (0.28)	0.002	13.8 (0.51)	0.002	14.0 (0.33)	0.002
**Hematocrit (%)**	25.64 (1.0)	56.22 (30.2)	0.375	30.5 (1.5)	0.031	63.63 (32.5)	0.003	65.9 (31.6)	0.002	79.63 (39.6)	0.003	41.64 (1.6)	0.002	42.94 (0.9)	0.003
**Platelets (×10^−3^)**	102.27 (13.04)	180.17 (33.14)	0.009	196.41 (32.72)	0.031	218.65 (34.03)	0.002	260.06 (38.09)	0.002	260.60 (28.20)	0.002	262.30 (24.71)	0.002	258.60 (19.98)	0.002
**Leucocytes (/mm^3^)**	2,266 (302.5)	2,873 (470.2)	0.018	5,123 (941.1)	0.015	5,426 (982.3)	0.001	6,239 (340.5)	0.001	6,441 (445.1)	0.002	6,996 (692.1)	0.002	6,539 (481.4)	0.002
**Neutrophils (/mm^3^)**	1,369 (381)	2,127 (636.3)	0.174	2,824 (932.4)	0.218	2,919 (844.4)	0.053	3,601 (369.9)	0.006	3,385 (306.8)	0.003	4,072 (463.9)	0.005	3,508 (355.6)	0.003
**Lymphocytes (/mm^3^)**	872.7 (110.5)	1,073 (145.2)	0.002	1,453 (192.5)	0.031	1,650 (143)	0.001	1,738 (177.1)	0.001	2,212 (275.1)	0.002	2,062 (282.4)	0.002	2,144 (265)	0.002
**Eosinophils (/mm^3^)**	45.3 (19.1)	76.8 (32.2)	0.125	202.8 (91.3)	0.250	237 (109.8)	0.062	429.9 (172.5)	0.031	388 (123)	0.125	320.6 (105.9)	0.125	308.8 (70.75)	0.062
**Monocytes (/mm^3^)**	280.2 (44.7)	443.8 (140.3)	0.320	523 (83.0)	0.109	554.3 (95.2)	0.024	458.2 (57.6)	0.067	491.7 (44.4)	0.039	492.5 (38.4)	0.037	527.2 (46.7)	0.013

Patients were evaluated on day 0 (D0), day (D7), day 14 (D14), day 21 (D21), day 30 (D30), day 60 (D60), day 90 (D90), and day 180 (D180) after the beginning of the treatment. Size increases of spleens and livers are expressed as the distances to the rib border in cm. All results are expressed as means + SE values. p-Values express significant differences between each treatment time and day 0 as disclosed by Wilcoxon matched-pairs rank test.

VL, visceral leishmaniasis.

### Cure of Visceral Leishmaniasis Is Associated With Increases in the Memory Multifunctional CD4^+^IL-2^+^TNF-α^+^IFN-γ^+^ and Effector CD4^+^TNF-α^+^IFN-γ^+^-Secreting T-Cell Populations

**Figure 1:**
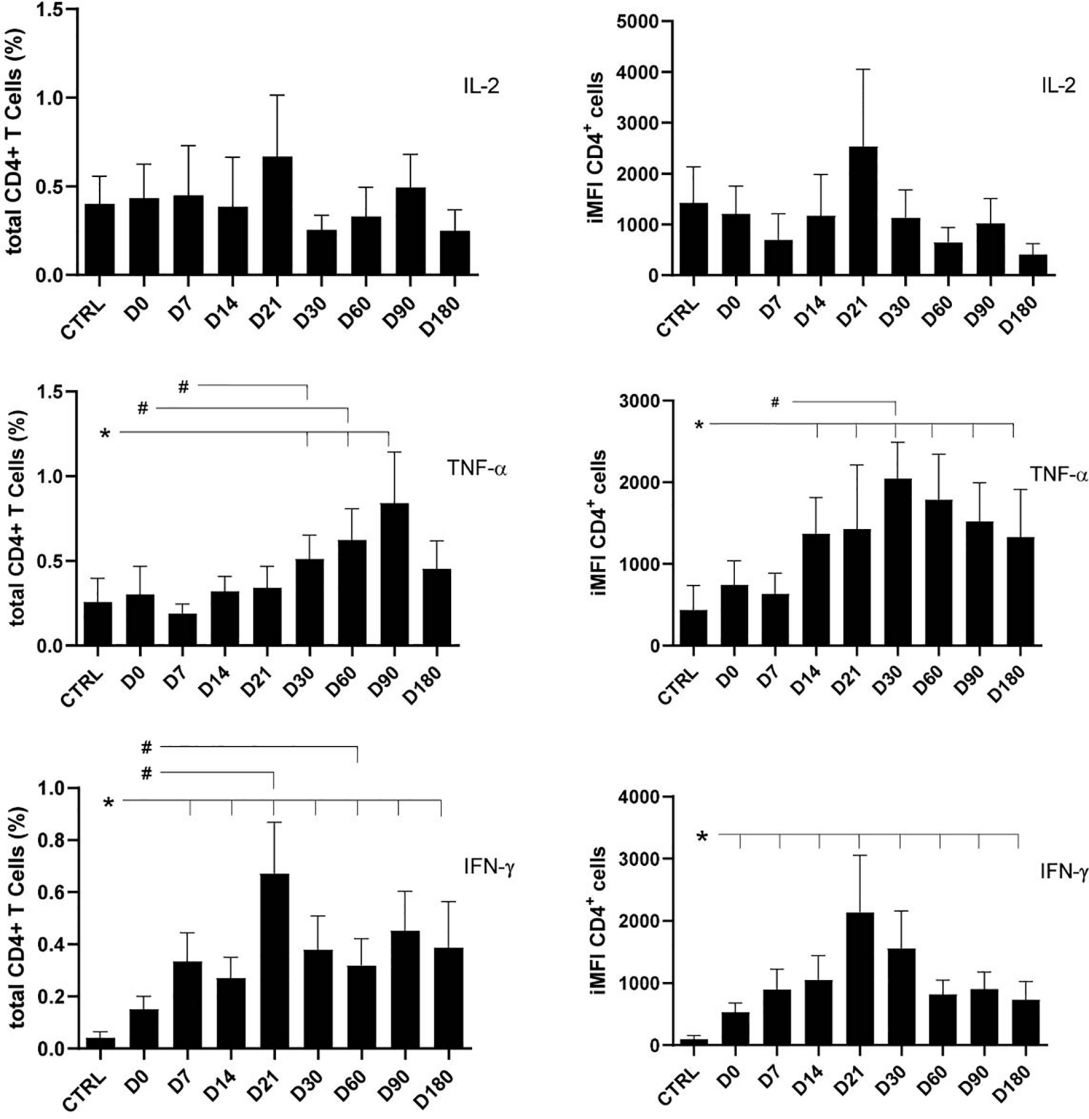
Total frequencies and integrated mean fluorescence intensity (iMFI) of cytokine-producing CD4^+^ T cells. The total frequencies and iMFI of each cytokine-expressing phenotype were determined in peripheral blood mononuclear cells (PBMCs) from healthy controls and patients before (D0) and during their treatment follow-up, after subtracting the background values corresponding to cells incubated without the soluble *Leishmania* antigen (SLA). Results in the panel are expressed as means + SE. Asterisks and horizontal lines indicate significant differences between times of treatments and healthy control records as disclosed by the Mann–Whitney method. Hashtags show significant differences between times of treatment as determined by the Wilcoxon matched-pairs rank statistical test.

The total frequencies and iMFI of IL-2-, TNF-α-, or IFN-γ-producing CD4^+^ T cells were assessed in patients with VL along their treatment (D0–D180) and in healthy controls (**Figure 1**). Stimulation of lymphocytes was performed with SLA. No significant variations among any treatment times or between treatment times and healthy controls were observed for IL-2 total frequencies or iMFI, suggesting that the basic production of IL-2 was preserved during treatment as in healthy controls (**Figure 1**).

In contrast, production of TNF-α increased along the treatment towards the cure on D180, when the frequencies of total producing CD4 T cells returned to normal levels, whereas iMFI still remained high. In fact, a rapid and time-dependent increase of TNF-α total frequency was observed starting from D7, reaching its maximum on D90, and decreasing to normal levels on D180. The iMFI of CD4^+^TNF-α producing T cells was, in contrast, still high on D180 (**Figure 1**).

However, the production of IFN-γ by CD4 T cells also increased alongside the healing process but started earlier than that of TNF-α (**Figure 1**), on D7, and reached a peak on D21, when both MA and LAMB therapy had finished. The intensity of IFN-γ production (iMFI), however, was already noted on D0 (**Figure 1**). In fact, the frequency of CD4^+^IFN-γ^+^ T cells on D21 was 4.7 times higher than on D0 (*p* = 0.016) and 16 times higher, if compared with healthy controls (*p* = 0.001) (**Figure 1**).

**Figure 2:**
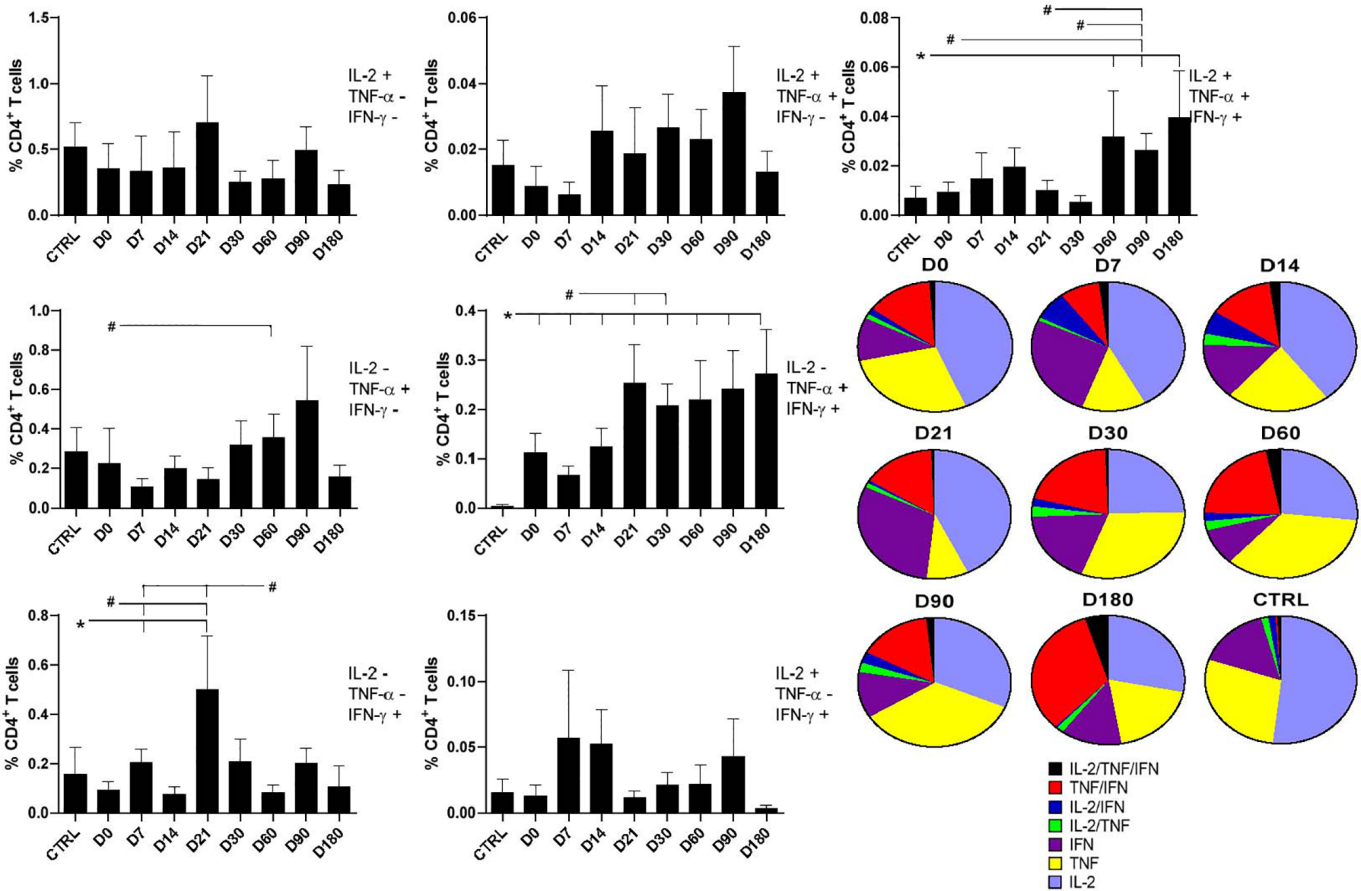
Distinct quality of CD4^+^ T in follow-up of visceral leishmaniasis (VL) treatment and of endemic control subjects. Evolution of the frequencies of CD4 T cells producing one cytokine (IL-2, TNF-α, or IFN-γ), and any combination of two or all three cytokines along the time. Each frequency was recorded after background subtraction of cells incubated without antigen [soluble *Leishmania* antigen (SLA)]. Results are expressed as means + SE, with asterisks and horizontal lines indicating significant differences between times of treatments and healthy controls as disclosed by the Mann–Whitney method. Hashtags show significant differences between times of treatment as determined by the Wilcoxon matched-pairs rank statistical test.

In addition, we used the multiparameter flow cytometry strategy for the analysis of the T-cell responses. Through this analysis, we are able to distinguish the frequency of CD4 T cells secreting only one (CD4^+^IL-2^+^, CD4^+^TNF-α^+^, and CD4^+^IFN-γ^+^), two (CD4^+^IL-2^+^TNF-α^+^, CD4^+^TNF-α^+^IFN-γ^+^, and CD4^+^IL-2^+^IFN-γ^+^), or three cytokines (CD4^+^IL-2^+^TNF-α^+^IFN-γ^+^) simultaneously (**Figure 2**).

As detected for total CD4^+^ cells producing IL-2 (**Figure 1**), all combinations of cells producing IL-2 were stable along the time and did not differ from those of healthy controls (**Figure 2**). In contrast, and as detected also for iMFI values and total frequencies of CD4 TNF-α-producing T cells (**Figure 1**), significant increases of the frequencies of CD4^+^ T cells producing only TNF-α^+^ were observed on D60 after treatment in comparison with D0. Furthermore, higher percentages of CD4^+^TNF-α^+^IFN-γ^+^ T cells were detected at all treatment times, if compared with healthy controls (**Figure 2**), and also there were higher percentages on D21 and D30, if compared with D0. The increase of the frequencies of CD4^+^TNF-α^+^IFN-γ^+^ T cells was impressive, and patients cured of VL showed a statistically significant enhancement of 78.9 times (*p* = 0.001) of this subpopulation if compared with healthy controls on D180 (**Figure 2**). In fact, the frequencies of CD4^+^TNF-α^+^IFN-γ^+^ T cells increased progressively in a time-dependent manner towards the cure, on D180. On the other hand, the frequencies of CD4^+^ T cells secreting only IFN-γ increased on D7 and D21 after the beginning of treatment, similar to what was detected for total frequencies and iMFI (**Figure 1**). However, they had decreased slightly by D60 (**Figure 2**). No significant variation was detected in the frequencies of CD4^+^IL-2^+^IFN-γ^+^ (**Figure 2**).

Finally, the frequencies of multifunctional CD4^+^IL-2^+^TNF-α^+^IFN-γ^+^ T cells increased significantly on D60, D90, and D180 as compared with those of controls, and on D90 as compared with those on D0, D30, and D21. Remarkably, on D180, the frequency of functional T cells was 5.7 times higher than that of healthy controls and 4.2 higher than in untreated patients (**Figure 2**), suggesting that the increase in multifunctional CD4^+^ T cells is correlated with cure. Our data suggest that the total response of CD4^+^ T cells promotes an increase in the quality of the Th1 response during the treatment, along with an increase in functional T-cell phenotypes (**Figure 2**).

The individual relative contribution of each cytokine-expressing phenotype population to the whole CD4^+^ T-cell response, for each treatment time, is summarized in the pie charts of **Figure 2**. The relative proportions of multifunctional CD4^+^IL-2^+^TNF-α^+^IFN-γ^+^ and the CD4^+^TNF-α^+^IFN-γ^+^-producing T cells increased along the treatment towards the cure, while in the cells of the healthy controls, the CD4^+^ T single-producer cells of IL-2^+^, TNF-α^+^, or IFN-γ^+^ were predominant. These results show that the treatment of VL determines the differentiation of CD4 T cells expected in the development or recovery of the Th1 response. In fact, in treated patients, besides the single-cytokine producers, double-cytokine producers are also present in minor proportions. In fact, the relative proportions of CD4^+^IL-2^+^TNF-α^+^ and CD4^+^IL-2^+^IFN-γ^+^ T cells increased until D14 and then decreased as the treatment advanced.

The evolution of the relative proportions of each CD4^+^ cytokine-expressing subtype along the treatment times is represented in **Supplementary Figure 2**. The relative proportions of cells producing only one cytokine decreased towards the end of treatment at D180, suggesting that the single producers converted into the double and triple producers, which reveals the recovery of the CD4 differentiation immune response. The frequencies of the double-cytokine producers CD4^+^IL-2^+^TNF-α^+^ remained high until D90, and those of the CD4^+^IL-2^+^IFN-γ^+^ cells were high on D7 and D14, but both decreased on D180. On the other hand, only the CD4^+^TNF-α^+^IFN-γ^+^ and the multifunctional T cells show a progressive time-dependent increase in frequencies along the treatment and reached their maximum values with the cure (**Supplementary Figure 2**). Our results suggest that the acquisition of a long-term memory T-cell CD4 response in combination with a strong effector-memory T-cell response is associated with the cure of VL.

### Cure of Visceral Leishmaniasis Is Associated With Increases in Memory Multifunctional CD8^+^IL-2^+^TNF-α^+^IFN-γ^+^, Single Producers of TNF-α, and Effector CD8^+^TNF-α^+^IFN-γ^+^ T-Cell Responses

**Figure 3:**
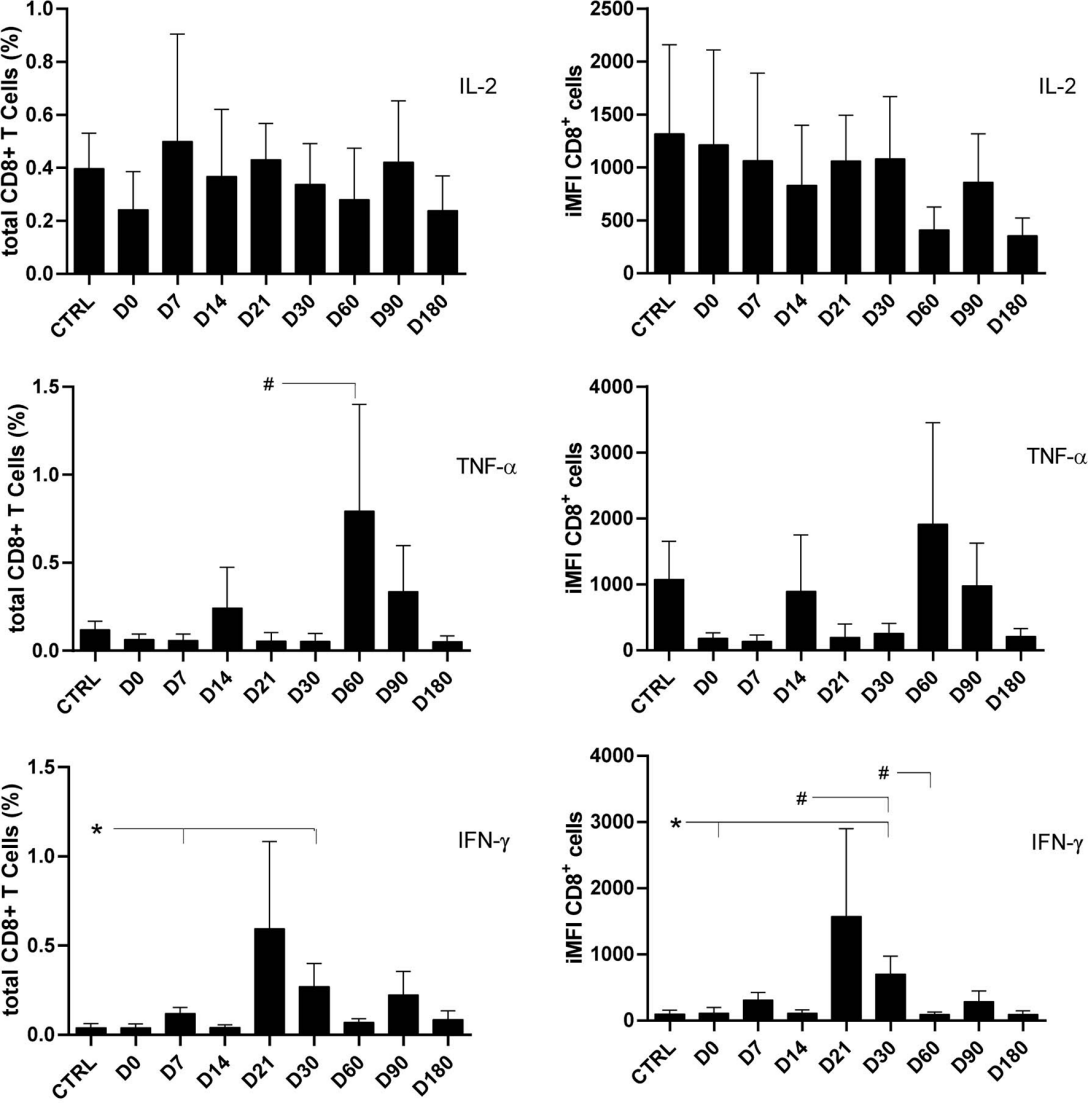
Total frequencies and integrated mean fluorescence intensity (iMFI) of cytokine-producing CD8^+^ T cells. The total frequencies and iMFI of each cytokine-expressing phenotype were determined in peripheral blood mononuclear cells (PBMCs) from healthy controls and patients before (D0) and during their treatment follow-up, after subtracting the background values corresponding to cells incubated without the soluble *Leishmania* antigen (SLA). Results in the panel are expressed as means + SE. Asterisks and horizontal lines indicate significant differences between times of treatments and healthy control records as disclosed by the Mann–Whitney method. Hashtags show significant differences between any time of treatment as determined by the Wilcoxon matched-pairs rank statistical test.

As previously described for CD4 T cells (**Figure 1**), the frequencies and iMFI of total IL-2-producing CD8^+^ T cells did not differ from those of controls at any of the treatment times, thus suggesting background basal and sustained stimuli of IL-2 in clonal expansion. In contrast, the proportions of total TNF-α^+^-secreting cells were 14 times higher on D60 than on D21, and the frequencies of total IFN-γ-secreting CD8^+^ lymphocytes and iMFI had increased six to seven times by D30 in comparison with controls and D0. A decrease in brightness was observed on D60 (**Figure 3**).

**Figure 4:**
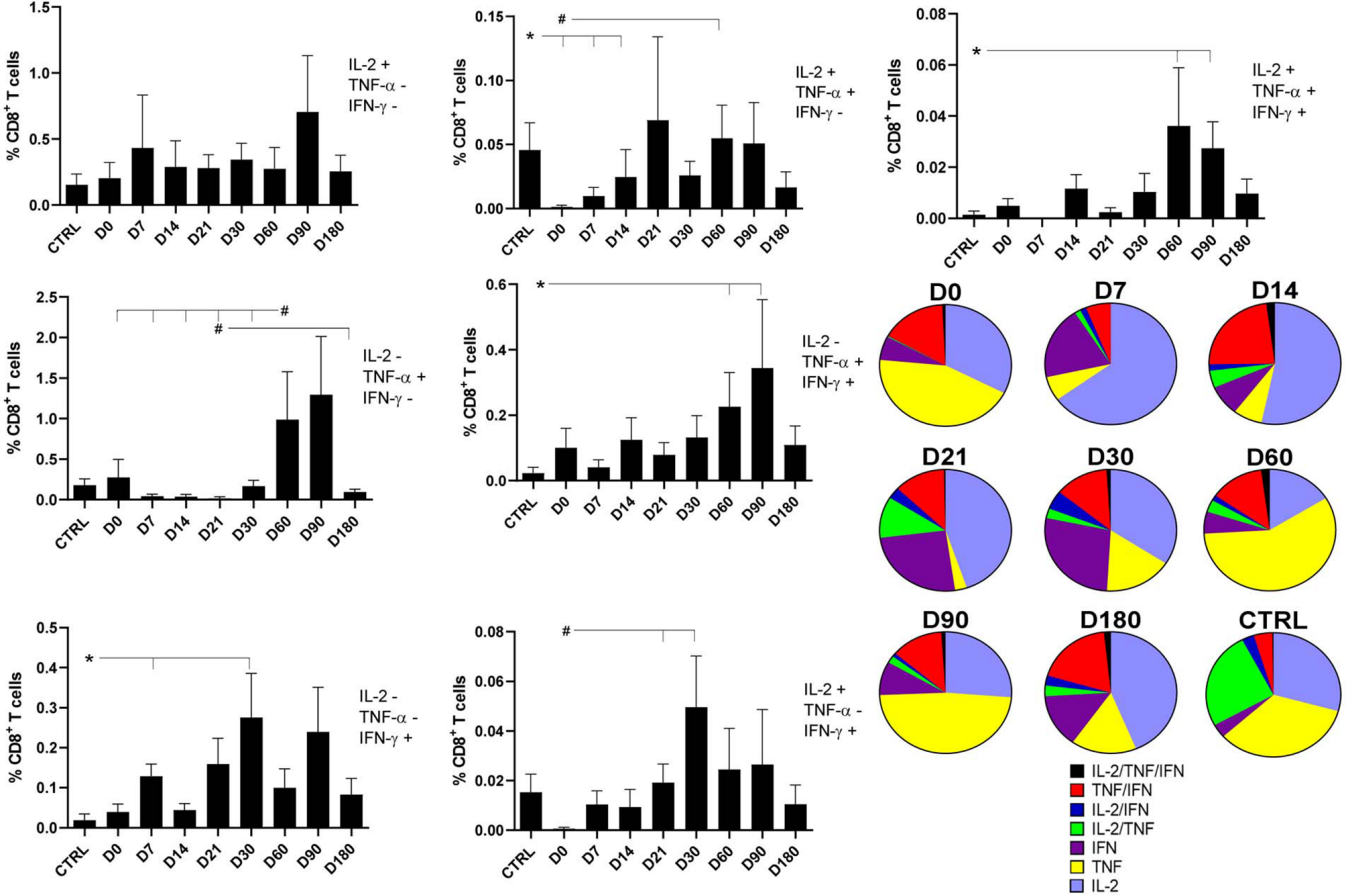
Distinct quality of CD8^+^ T in follow-up of visceral leishmaniasis (VL) treatment and of endemic control subjects. Evolution of the frequencies of CD8^+^ T cells producing one cytokine (IL-2, TNF-α, or IFN-γ), and any combination of two or all three cytokines along the time. Each frequency was recorded after background subtraction of cells incubated without antigen [soluble *Leishmania* antigen (SLA)]. Results are expressed as means + SE, with asterisks and horizontal lines indicating significant differences between times of treatments and healthy controls as disclosed by the Mann–Whitney method. Hashtags show significant differences between times of treatment as determined by the Wilcoxon matched-pairs rank statistical test.

As observed for the CD4^+^ T cells (**Figures 1** and **2**) and for the CD8^+^ total frequencies and iMFI (**Figure 3**), the multiparameter analysis confirmed that the frequencies of the IL-2 single producers CD8^+^ T cells did not change along the time (**Figure 4**), whereas the frequencies of single producers of TNF-α were 3.6 times higher on D60 than on D0. Furthermore, the frequency of single producers of IFN-γ was 14.6 times greater on D30 as compared with healthy controls.

Regarding the CD8^+^ T cells producing two cytokines, the frequencies of CD8^+^IL-2^+^TNF-α^+^ T cells had increased by 41.2 times by D60 compared with D0 (**Figure 4**), and the frequencies of CD8^+^TNF-α^+^IFN-γ^+^ T cells had increased by 9.6 times on D60 and by 14.7 times on D90 as compared with controls. In contrast, and as detected for single producers of IFN-γ, an earlier rise of the proportions of the CD8^+^IL-2^+^IFN-γ^+^ T cells was observed with increases of 28.2 and 81.4 times, on D21 and D30, respectively, when compared with D0 (**Figure 4**).

However, similar to what was described for CD4 T cells (**Figure 2**), the proportions of the multifunctional CD8^+^IL-2^+^TNF^+^IFN-γ^+^ T cells increased significantly, if compared with controls, on D60 and D90, suggesting the involvement of memory T cells in the cure process (**Figure 4**).

The pie chart shows that soon after the beginning of the treatment, on D14, the frequencies of double producers of TNF-α^+^IFN-γ^+^ and IL-2^+^TNF-α^+^ lymphocytes start to increase, indicating the expected evolution of the CD8 *Leishmania-*specific T-cell response. After cure, on D180, the proportion of multifunctional memory T cells is at a maximum. As predicted by Seder’s model (26), the multifunctional central-memory CD8^+^ T cells seem to interconvert with the TNF-α^+^IFN-γ^+^ effector-memory T cells during the treatment. Furthermore, as expected from Seder’s model, when cure is achieved by D180, both types of cells are present in high proportions, and there is also an increase in the frequencies of the single producers of IFN-γ, which are considered terminal effector CD8 T cells (**Figure 4**).

An increase in the quality of the CD8^+^ T cells response is observed when there is a concomitant increase of frequencies of the central-memory (multifunctional CD8^+^IL-2^+^TNF-α^+^IFN-γ^+^), effector-memory T cells (CD8^+^TNF-α^+^IFN-γ^+^), and terminal effector CD8 T cells (CD8^+^IFN-γ^+^) (**Figure 4**).

The evolution of the relative proportions of each CD8^+^ cytokine-expressing phenotype along the time of treatment is represented in **Supplementary Figure 3**. Confirming the data of **Figure 4**, healthy controls individuals always showed lower proportions of the single producers of the IFN-γ^+^ terminal effector, and double producers of the TNF-α and IFN-γ effector memory CD8 T cells compared with VL patients during the treatment. In agreement, except for those at D7, the multifunctional CD8^+^IL-2^+^TNF-α^+^IFN-γ^+^-TCM cells showed frequencies above those of healthy controls during the whole treatment and reached maximum values at the cure (D180) (**Supplementary Figure 3**).

### Increase of CD8^High^ and Decrease of CD8^Low^ Phenotypes Correlate With Visceral Leishmaniasis Cure

**Figure 5:**
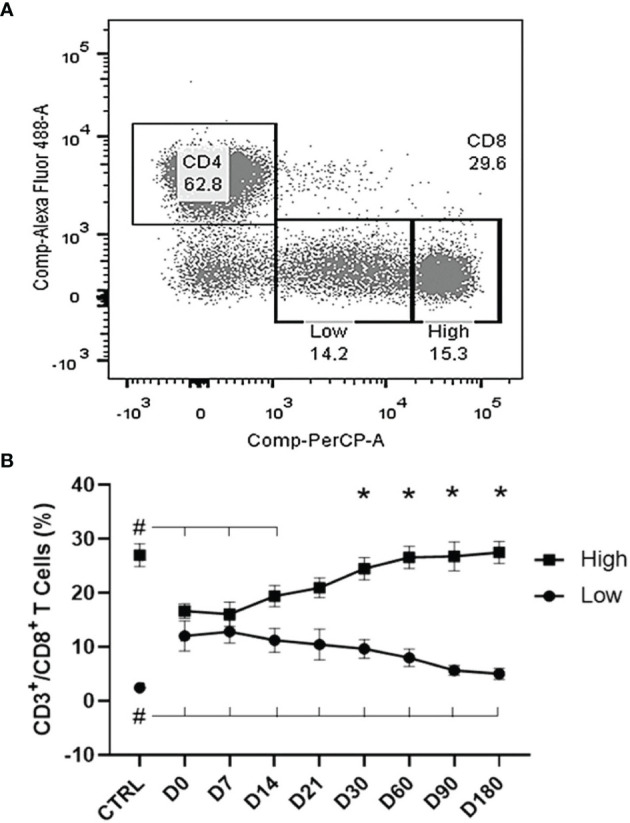
Evolution of CD8^Low^ and T CD8^High^ T-cell populations. **(A)** Two subpopulations were gated within the CD8^+^ T cells according to their brightness intensity: CD8^High^ and CD8^Low^ in a visceral leishmaniasis (VL) patient before treatment. **(B)** Increases of CD8^High^ and decreases of CD8^Low^ T-cell frequencies along the treatment. Results are expressed as means ± SE values. The Shapiro–Wilk test disclosed that the distribution of frequencies was normal. Asterisks show the significant variations between CD8^High^ and CD8^Low^ populations starting from day 30, as disclosed by the two-way ANOVA Sidak analysis. Hashtags indicate significant differences between each time of treatment and healthy controls values as disclosed by Student’s *t*-test.

Two different subpopulations of CD8^+^ T cells were identified by the flow cytometry analysis, according to their brightness intensity. Cells showing a higher expression of CD8^+^ molecules were classified as CD8^High^, while those showing a lower expression were considered as CD8^Low^ (**Figure 5A**). The differences between the two populations were highly significant starting from D30 (*p* < 0.0001) (**Figure 5B**). CD8^High^ frequencies were high in healthy controls and impressively low in untreated patients (D0 and D7). However, they progressively recovered to normal values starting from D21 and reached the highest values at the cure, on D180 after the beginning of the treatment (**Figure 5B**). In contrast, the frequencies of CD8^Low^ cells were higher in untreated patients than in healthy controls at D0, and these decreased along the treatment and did not recover to their normal values even at D180. In fact, the evolution of the frequencies of CD8^High^ and CD8^Low^ phenotypes was negatively correlated (R = −0.676; *p* < 0.0001). Our results indicate that the progress of chemotherapy treatment goes along with an increase of CD8^High^ and a decrease of CD8^Low^ frequencies that represent markers of the cure for VL.

### Correlations Between Clinical Outcomes and T-Cell Phenotypes

In VL, an increase in the size of the spleen and decrease in hematocrit, hemoglobin, monocyte, and leukocyte counts are typical markers of this disease (**Table 1**). We observed that during the treatment, the frequencies of CD8 single producers of TNF-α and of CD8^High^ correlated negatively with spleen sizes, while the frequencies of CD8^Low^ T cells correlated negatively with the leukocyte and monocyte counts, indicating that CD8 single producers of TNF-α and of CD8^High^ are strong correlates of cure, while CD8^Low^ is a marker of disease (**Table 2**).

**Table 2:** Correlation between clinical outcomes and CD4^+^ and CD8^+^ T-cell phenotypes.

T-cell phenotypes	Clinical outcome	*R* values	*p*-Values
CD4^+^IL-2^+^ total frequencies	Monocytes	0.271	0.0212
CD4^+^TNF-α^+^ total frequencies	Hemoglobin	0.336	0.0041
	Hematocrit	0.276	0.0198
CD4^+^IFN-γ^+^ total frequencies	Monocytes	0.383	0.0009
CD4^+^IFN-γ^+^ iMFI	Monocytes	0.352	0.0024
CD4^+^IL-2^+^TNF-α^−^IFN-γ^−^	Monocytes	0.293	0.0142
CD4^+^IL-2^−^TNF-α^+^IFN-γ^−^	Monocytes	0.273	0.0228
CD4^+^IL-2^−^TNF-α^−^IFN-γ^+^	Monocytes	0.264	0.0279
CD4^+^IL-2^+^TNF-α^−^IFN-γ^+^	Monocytes	0.283	0.0168
CD4^+^IL-2^+^TNF-α^+^IFN-γ^+^	Monocytes	0.331	0.0048
CD8^+^IL-2^−^TNF-α^+^IFN-γ^−^	Spleen size	−0.306	0.0093
	Hemoglobin	0.262	0.0267
	Neutrophils	0.287	0.0154
CD8^+^IL-2^+^TNF-α^+^IFN-γ^+^	Hemoglobin	0.334	0.0041
	Hematocrit	0.249	0.0357
CD8^High^	Spleen size	−0.325	0.0053
	Hemoglobin	0.308	0.0084
	Hematocrit	0.270	0.0224
	Leukocytes	0.501	<0.0001
	Neutrophils	0.442	<0.0001
	Lymphocytes	0.262	0.0259
	Monocytes	0.348	0.0027
	Eosinophils	0.407	0.0015
CD8^Low^	Leukocytes	−0.257	0.0291
	Monocytes	−0.368	0.0014
MA CD4^+^IFN-γ^+^ total frequencies	Liver size	−0.344	0.0499
	Monocytes	0.432	0.0120
MA CD4^+^IFN-γ^+^ iMFI	Liver size	−0.348	0.0467
	Monocytes	0.381	0.0286
MA CD4^+^TNF-α^+^ total frequencies	Hemoglobin	0.364	0.0371
	Hematocrit	0.369	0.0245
	Platelets	0.387	0.0258
	Leukocytes	0.487	0.0040
	Neutrophils	0.458	0.0073
MA CD4^+^TNF-α^+^ iMFI	Liver size	−0.472	0.0055
	Spleen size	−0.566	0.0006
	Platelets	0.358	0.0404
	Leukocytes	0.418	0.0155
MA CD8^+^IFN-γ^+^ total frequencies	Platelets	−0.388	0.0496
LAMB CD4^+^IL-2^+^ total frequencies	Lymphocytes	−0.274	0.0427
LAMB CD4^+^IL-2^+^ iMFI	Eosinophils	0.316	0.0412
LAMB CD8^+^IL-2^+^ iMFI	Lymphocytes	−0.274	0.0450

Correlation analysis between variables was performed using Spearman’s two-tailed method.

LAMB, liposomal amphotericin B; MA, meglumine antimoniate; iMFI, integrated mean fluorescence intensity.

In addition, the total frequencies of CD4^+^TNF-α^+^ T cells, the frequencies of CD8 triple secretors of IL-2, TNF-α, and IFN-γ and of CD8^High^ T cells correlated positively with the increases of hematocrit and hemoglobin counts, while the frequency of CD8 single producer of TNF-α is associated only with the increase in hemoglobin counts (**Table 2**). Furthermore, during the treatment, the increase of monocyte counts was positively correlated with the increases of the CD4^+^IL-2^+^, the CD4^+^IFN-γ^+^ total frequencies, and CD4^+^IFN-γ^+^ iMFI. Monocyte frequencies also correlated with the increases of the frequencies of the CD4^+^ T cells as single producers of IL-2, TNF-α, and IFN-γ; double producers of IL-2 and IFN-γ; and triple secretors of IL-2, TNF-α, and IFN-γ, and with the frequencies of CD8^High^ T cells (**Table 2**). The frequencies of the CD8 single producers of TNF-α correlated positively with neutrophils, while CD8^High^ correlated with the counts of leukocytes, neutrophils, lymphocytes, and eosinophils (**Table 2**). Thus, these variables indicate that the recovery of the T CD4 response can also be considered as a marker of the cure. In contrast, only the increases in the frequencies of CD8 single secretors of TNF-α or triple secretors are associated with a decrease of symptoms. Remarkably, the increases of CD8^High^ and the decreases of CD8^Low^ frequencies are definitively strong correlates of cure.

**Figure 6:**
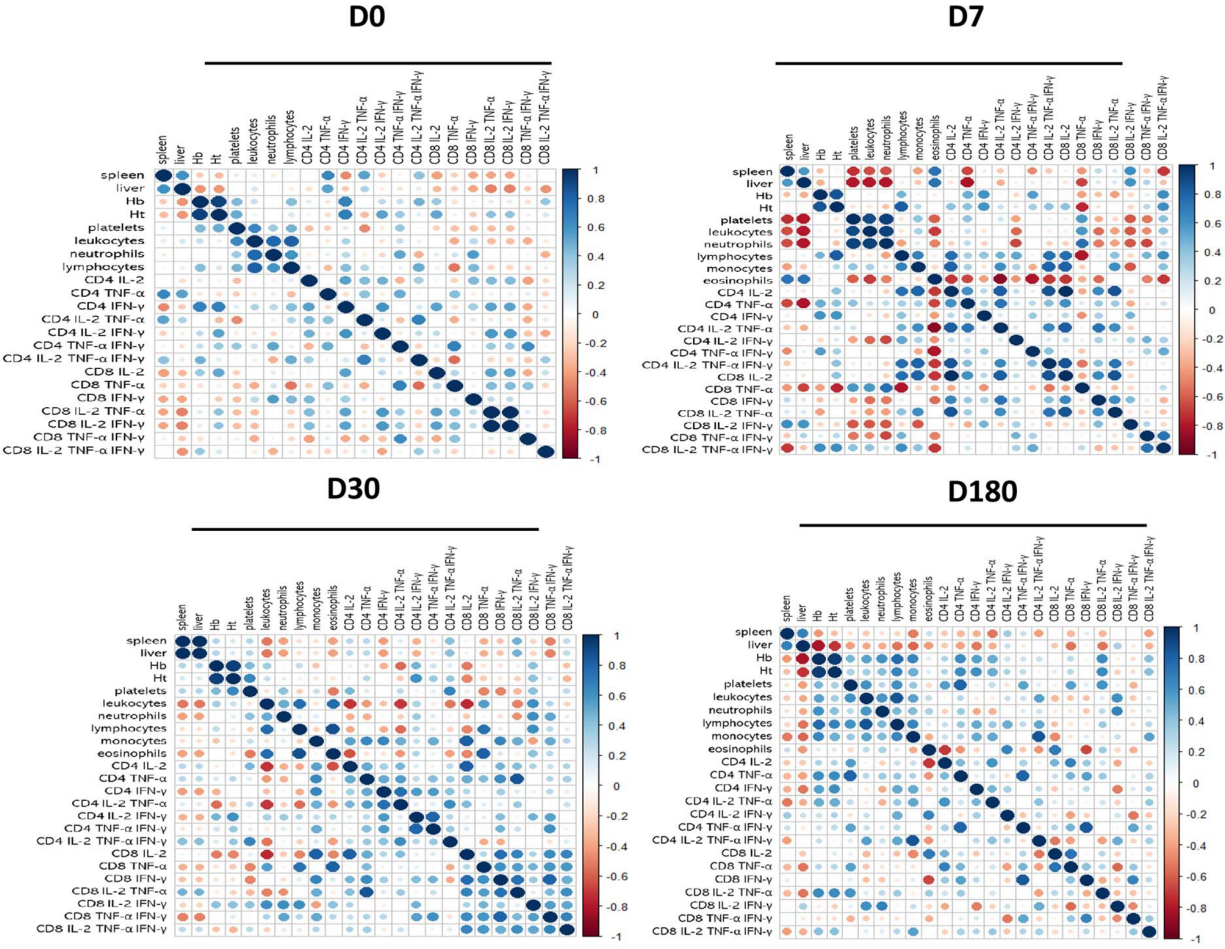
Correlation matrix heatmap of clinical outcomes and cytokine combinations of CD4 and CD8 T cells. Spearman’s correlation coefficient was used to compute the relevance and redundancy of the features. Blue dots represent positive correlations, and red dots are negative Spearman’s correlation.

Spearman’s correlation matrix heatmap demonstrated the association of the profiles between the clinical outcomes, hematological counts, and the cytokine-producing CD4 and CD8 T cells, for each treatment time separately. We also showed the corrplots for D0, D7, D30, and D180 (**Figure 6**). The correlation patterns between all these factors changed gradually during the treatment. For instance, on D180, when the patient was considered completely cured, both liver and spleen sizes were negatively associated (red dots) with the frequencies of CD4^+^IL-2^+^, CD4^+^TNF-α^+^, CD4^+^IFN-γ^+^, CD4^+^IL-2^+^TNF-α^+^, CD8^+^TNF-α^+^, CD8^+^IL-2^+^TNF-α^+^, and CD8^+^IL-2^+^TNF-α^+^IFN-γ^+^ T cells. In addition, the spleen sizes were also negatively associated with the frequencies of CD4^+^IL-2^+^TNF-α^+^IFN-γ^+^, and the liver sizes were negatively correlated to the proportions of CD4^+^TNF-α^+^IFN-γ^+^, CD8^+^IL-2^+^, and CD8^+^TNF-α^+^IFN-γ^+^. Notably, on D180, the CD4^+^TNF-α^+^IFN-γ^+^ T cells correlated strongly positively with leukocytes, lymphocytes, and monocytes and weakly positively with hemoglobin, platelets, and neutrophils. This analysis confirms that the complete development and differentiation of the CD4 and CD8 *Leishmania*-specific T cells is associated with the healing process and that these T-cell phenotypes are strong correlated with VL cure.

### The Chemotherapy Treatments With Meglumine Antimoniate or Liposomal Amphotericin B Promote the Recovery of the T Cell Immune Responses

In VL, the immune response is directly affected by the drugs that act mainly to improve the clinical outcome. Therefore, we analyzed the chemotherapeutic effect of LAMB and of MA separately. Patients who exhibited more severe symptoms associated with age, comorbidities, unexpected hemophagocytosis, relapse, etc. were treated with LAMB, whereas less severe cases received MA.

**Figure 7:**
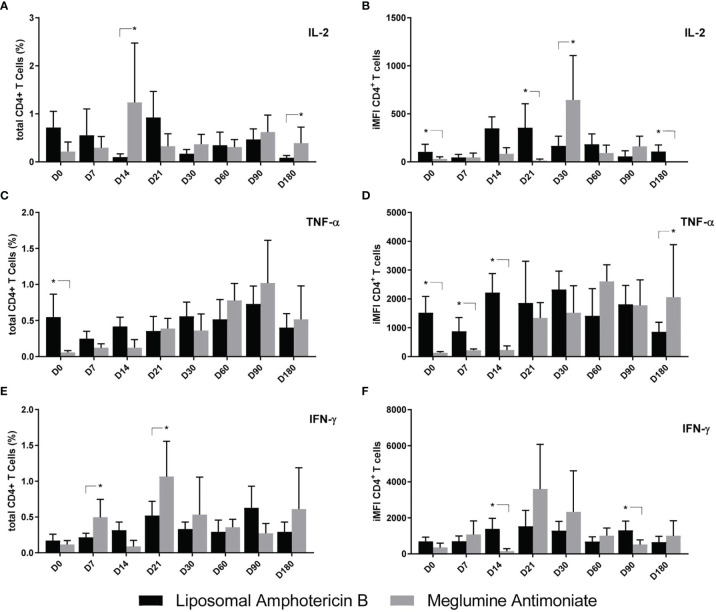
Cytokine production by CD4^+^ T cells of patients treated with meglumine antimoniate (Glucantime^®^) and amphotericin B Frequency **(A–C)** and integrated mean fluorescence intensity (iMFI) **(D–F)** of T CD4 cells producing IL-2, TNF-α, and IFN-γ from patients treated with Glucantime^®^ (N = 8) and amphotericin B (N = 5). The frequencies and iMFI of each cytokine expressing phenotype were recorded after background subtraction of cells incubated without antigen [soluble *Leishmania* antigen (SLA)]. Results in the panel are expressed as means + SE. Asterisks and horizontal lines indicate statistical differences between treatments as disclosed by IC95%.

The total frequencies and iMFI of cytokine producers of CD4^+^ T cells are represented in **Figure 7**. Before treatment, on D0, the levels of iMFI-IL-2 and total frequencies and iMFI-TNF-α^+^ CD4^+^ T cells were higher in patients treated with LAMB (**Figures 7B–D**). In contrast, the total frequencies of CD4^+^IL-2 response were 12.4 and 4.5 times higher on D14 and D180, respectively; and the iMFI IL-2 response was 3.9 higher on D30 in patients treated with MA (**Figures 7A, B**). The TNF-α iMFI response remained higher on D7–D14 in patients treated with LAMB but increased towards the cure only in patients treated with MA (**Figure 7D**). Additionally, the total frequencies of IFN-γ-secreting CD4^+^ T cells were 2.3 and 2.0 times higher in patients treated with MA, on D7 and D21, respectively (**Figure 7E**). Our results suggest that the MA treatment stimulates higher secretions of IL-2 and TNF-α along the treatment and up to the cure, and of IFN-γ until D21. Treatment with LAMB, on the other hand, had no impact on the IL-2 iMFI response and decreased the frequencies of total TNF-α-secreting T cells along time.

**Figure 8:**
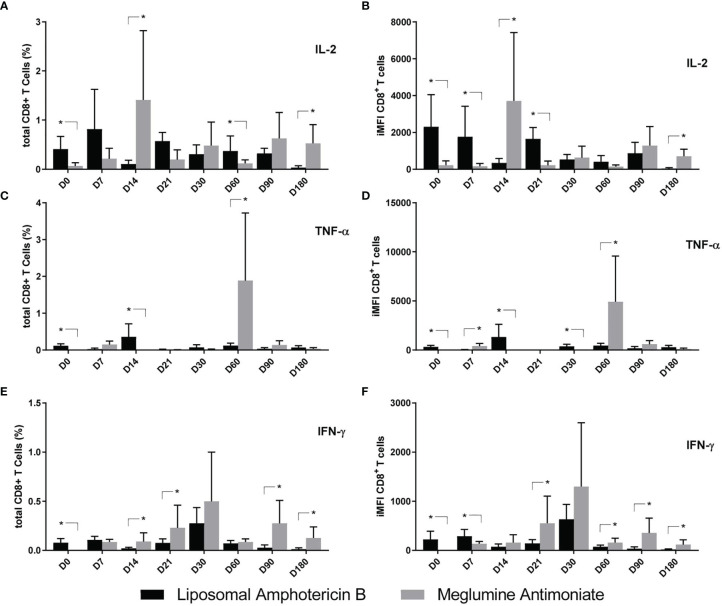
Cytokine production by CD8^+^ T cells of patients treated with meglumine antimoniate (Glucantime^®^) and amphotericin B Frequency **(A–C)** and integrated mean fluorescence intensity (iMFI) **(D–F)** of T CD4 cells producing IL-2, TNF-α, and IFN-γ from patients treated with Glucantime^®^ (N = 8) and amphotericin B (N = 5). The frequencies and iMFI of each cytokine expressing phenotype were recorded after background subtraction of cells incubated without antigen [soluble *Leishmania* antigen (SLA)]. Results in the panel are expressed as means + SE. Asterisks and horizontal lines indicate statistical differences between treatments as disclosed by IC95%.

As for the CD8^+^ T-cell response, and also in agreement with their higher severity, the levels of iMFI and total frequencies of IL-2^+^, TNF-α^+^, and IFN-γ^+^-secreting T cells on D0 were higher in the patients who were going to receive treatment with LAMB (**Figures 8A–F**). Cytokine secretion was predominant in LAMB-treated patients mainly until D7 (**Figures 8D, F** **)** or D14 (**Figures 8C, D** **)**. On the other hand, MA treatment determined 10.6–16.1 times higher responses of IL-2 on D14 and D180 (**Figures 8A, B** **)**; 15–10.6 higher responses of TNF-α on D60 (**Figures 8C, D** **)**; and 3–10.1 times higher responses of IFN-γ on D21, D90, and D180 (**Figures 8E, F** **)**. We conclude that the treatment with MA generated long-lasting predominant responses of cytokine secretion. More than the CD4^+^ T cells, the IL-2 and IFN-γ-secreting CD8 T-cell predominance appears to be associated with the cure.

Additionally, we analyzed the correlations between the clinical outcomes and the frequencies and iMFI of cytokine-secreting responses, considering the patients treated with MA or LAMB separately (**Table 2**). More important clinical parameters of cure were associated with immunological variables in patients treated with MA. In fact, the CD4^+^ IFN-γ^+^ total frequencies and iMFI of MA treated patients increased as their liver sizes and monocyte counts decreased. The size of the livers is an important hallmark of VL. Furthermore, the intensity of TNF-α secretion by CD4^+^ T cells was highly correlated with the decrease of liver and spleen sizes and with the increase in platelets and leukocyte counts. The correlations with the decrease of spleen and liver sizes exhibited the highest *R* values. In addition, and importantly, the total frequencies of CD4^+^TNF-α^+^ were positively correlated to the hemoglobin and hematocrit values and the platelet, leukocyte, and neutrophil counts. The intensity of the secretion of TNF-α by CD4^+^ T cells also increases in correlation to platelets and leukocytes. In contrast, the total frequencies of CD8^+^IFN-γ^+^ of MA-treated patients were negatively correlated to the platelet counts (**Table 2**). This result might be an indication of the association of terminal effector CD8^+^ T cells in VL.

In contrast, only three variables were associated with the clinical signs in patients treated with LAMB. The total frequencies of CD4^+^IL-2^+^ and iMFI of CD8^+^IL-2^+^ cells correlated negatively with lymphocyte counts, while the CD4^+^IL-2^+^iMFI values were correlated with eosinophils. However, studies with larger cohorts of patients are needed to confirm this hypothesis.

In order to clarify the impact of the MA and LAMB treatments on the immune responses, we further analyzed the frequencies of cytokine-expressing CD4^+^ T cells, but considering separately the patients treated with LAMB or MA (**Supplementary Figure 4**). Similarly to what was observed when all patients were considered together (**Figures 1** and **2**), no variation of frequencies of single secretors of IL-2 was detected. In contrast, and as detected for the analysis of the total frequencies (**Figure 7**), the MA treatment promoted a main pronounced response in CD4 T cells, which secrete IFN-γ. In fact, the MA effect was observed in the increase of the frequencies of terminal-effector single secretors of IFN-γ throughout the whole treatment and until D180, of double secretors of IFN-γ and IL-2 on D7 and D14, and of the multifunctional CD4^+^IL-2^+^TNF-α^+^IFN-γ^+^ on D7. MA also induced increased proportions of double secretors of IL-2 and TNF on D14. In contrast, LAMB treatment impacted more prominently the secretion of TNF-α by single secretors on D7 and D90; by double secretors of IL-2 and TNF-α on D21, D30, and D180; by double secretors of TNF-α and IFN-γ on D14; by double secretors of IL-2 and IFN-γ on D21 and D60; and by multifunctional CD4^+^IL-2^+^TNF-α^+^IFN-γ^+^ on D30 and D60.

Additionally, the treatment with MA showed a stronger impact on the development of the CD8^+^ cytokine-secreting T-cell response (**Supplementary Figure 5**), by increasing the frequencies of single producers of IL-2 on D14, D30, and D180; of single producers of TNF-α on D21 and D60; of single producers of IFN-γ on D14 and D21; of double producers of IL-2 and TNF-α by D14 and D60; of double producers of TNF-α and IFN-γ by D7 and D21; and of double producers of IL-2 an IFN-γ by D14, D90, and D180. In contrast, LAMB treatment enhanced the frequencies of single producers of IL-2 on D7; of single producers of TNF-α on D14; of double producers of IL-2 and TNF-α by D7 and D21; of double producers of TNF-α and IFN-γ by D14, D90, and D180; and of multifunctional CD8^+^IL-2^+^TNF-α^+^IFN-γ^+^ on D14, D21, D30, and D180.

Therefore, and different from what was suggested by the analysis of the total frequencies and iMFI (**Figures 7** and **8**), the detailed analysis of the cytokine responses disclosed that both the MA and LAMB therapies promote immune responses. MA treatment had more impact on the secretion of IFN-γ, LAMB had stronger influence on the secretion of TNF-α by CD4^+^ T cells, but both generated memory CD4^+^ T-cell responses. In addition, the therapy with MA promoted increased frequencies of all types of single- and double-cytokine secretors, but only LAMB promoted higher responses of multifunctional CD8^+^IL-2^+^TNF-α^+^IFN-γ^+^ T cells.

## Discussion

In this work, we aimed to elucidate if the cure of VL with chemotherapy is associated with the differentiation of the CD4^+^ and CD8^+^ cytokine-expressing T cells (26). First, we analyzed the evolution of the clinical signs of VL along the treatment. In agreement with other results in the literature (11, 32, 34), the drug treatment reduced the sizes of the spleens and livers and increased the hematocrit and hemoglobin counts soon after the beginning of chemotherapy, but cure was only considered complete at 180 days after the beginning of the treatment.

The participation of CD4^+^ T cells in VL has been widely addressed. These cells were found to be associated with resistance to infection, as they can produce different types of cytokines linked to the Th1, Th2, or Th17 cell responses (11, 35–37). In this study, we observed an increase in the frequencies of CD4^+^ T cells producing TNF-α throughout the treatment. TNF-α is one of the main cytokines involved in protection against VL; however, at high levels, it can also cause tissue damage (38, 39). On the other hand, the IFN-γ-producing CD4^+^ T cells are considered one of the main functional cells during *Leishmania* infection (13, 40). However, IFN-γ promotes macrophage stimulation and inhibition of Th2 cells; thus, when its secretion increases, the patient immune response may be more effective against the parasite (35).

In this study, we described for the first time the functional profile of CD4^+^ and CD8^+^ T cells regarding their production of IL-2, TNF-α, and IFN-γ, before and throughout treatment of VL patients. While IL-2 secretion by CD4^+^ lymphocytes remained stable, the TNF-α secretion increased after 30 days of treatment and reached maximum values on D90, while the IFN-γ secretion showed a peak on D21 and a decreased towards the cure on D180. In agreement with TNF-α, the frequencies of CD4^+^ double secretors of TNF-α and IFN-γ increased as of D21 until D180, indicating that this phenotype is strongly associated with the progress to the cure. Furthermore, the analysis of the relative proportions of the phenotypes and their evolution along the time confirmed the importance of the CD4^+^TNF-α^+^IFN-γ^+^ lymphocytes, which combine both the effector and memory functions of the Th1 response cells (26). In addition, the proportions of multifunctional CD4^+^IL-2^+^TNF-α^+^IFN-γ^+^ T cells, which concentrate the optimized central-memory (TCM) and effector memory (TEM) functions, increased through to the end of the monitoring period on D90. Our results highlighted the involvement of TNF-α and IFN-γ in the process of cure. In agreement, it was shown that neutralization of IFN-γ and TNF-α promotes an IL-4 production (40).

Although PBMCs of VL patients are usually described as non-responders to leishmanial antigens, the CD4^+^ T cells are the main source of *Leishmania*-specific-IFN-γ secretion during VL, as detected by the whole-blood stimulation assay (41). However, this production of IFN-γ was decreased by the impairment of the (HLA)-DR antigen, suggesting that it is mediated by a typical HLA-TCR receptor association. Moreover, and in spite of the progressive immunosuppression of VL, an intrinsic IFN-γ produced by patients helped to control the expansion of the parasites (41), and the lack or diminished production of IFN-γ determines the patient vulnerability to VL (42). On the other hand, the IL-6 cytokine, which is associated with severity and death of VL, was related to the impairment of TNF-α secretion soon after infection and to the consequent inhibition of Th1 response (36, 42, 43). In fact, TNF-α is associated with the activation of effector immune responses (42), and the TNF-α levels only returned to normal after the anti-*Leishmania* therapy, which correlated with clinical cure (44).

TCM cells are found in the T-lymphocyte areas of secondary lymphoid organs, where they can proliferate and transform into TEM cells, which show reduced expression of the CCR7 marker, and high or low expression of CD62L marker, and can migrate to injured or inflamed tissues (25). In this investigation, the cure of human VL was associated with increases of the frequencies of the antigen-specific CD4^+^IL-2^+^TNF-α^+^IFN-γ^+^ T cells, which are considered to be memory T cells (TCM), and of the effector CD4^+^TNF-α^+^IFN-γ^+^-secreting T cells, called TEM cells. Enhanced frequencies of antigen-specific TCM T cells were also found in cured and asymptomatic VL patients in India (45) and in cured patients of tegumentary leishmaniasis caused by *Leishmania* (*V.*) *braziliensis* in Brazil (46). In our study, the frequencies of *Leishmania*-specific CD4^+^IL-2^+^TNF-α^+^IFN-γ^+^ T cells and their relative proportions among the other phenotypes increased significantly towards the cure, on days 60, 90, and 180. In cured patients, these cells correlate negatively with the sizes of the spleens and positively with the hemoglobin, platelet, leukocyte, neutrophil, lymphocyte, and monocyte counts. Thus, as expected, an increase in the multifunctional TCM CD4^+^IL-2^+^TNF-α^+^IFN-γ^+^ population is a strong correlate and marker of cure. In agreement with our results, Macedo et al. (46) observed that the crude lysate of *L.* (*V.*) *braziliensis* increased the frequencies of the CD4^+^IL-2^+^TNF-α^+^IFN-γ^+^ T cells in PBMC of cured patients to 28%, whereas the crude antigen of *Leishmania* (*L.*) *amazonensis* increased the frequencies of single producers of IFN-γ to 57%.

This is the first report to describe the evolution of the cytokine-secreting CD4 T-cell response during 6 months of treatment and monitoring to the cure of human VL. In a previous work, we also observed increased frequencies of CD4^+^IL-2^+^TNF-α^+^IFN-γ^+^ T cells, in response to the NH36, F2, and crude lysate antigen in cured VL patients and asymptomatic DTH+ subjects, as well as increased proportions of CD4^+^TNF-α^+^IFN-γ^+^ effector T cells, in response to NH36, F1, and F2 antigens of cured and untreated patients (11).

In contrast to our results, and to the prediction that considers the CD4^+^ IL-2^+^TNF-α^+^IFN-γ^+^ T cells as a correlate to protection (27), in *Leishmania* (*L.*) *major* infected soldiers, these cells together with CD4^+^TNF-α^+^IFN-γ^+^ T lymphocytes were the most frequent phenotypes (47). After treatment, these frequencies were significantly lower (47). However, as demonstrated in experimental models, after the elimination of the parasites, the central-memory CD4 T cells remain responsible for the resistance to reinfection (48). The CD4^+^ long-term effector memory responses throughout the treatment against VL might thus be related to a protective role against re-exposure to the parasite. In fact, in our study, the long-term memory response, represented by these multifunctional CD4^+^ T cells, started mainly towards the end of the treatment, on D60.

Several studies have indicated that the health and disease processes depend on dynamic immune responses that alter the frequency and quality of antigen-specific T cells (49–51). Fewer studies have investigated the role of CD8 T cells in human VL (19, 21). Although increased frequencies of antigen-specific CD8 T cells have been found in cured patients (21), the exhaustion of CD8 T cells have also been reported, probably due to changes in the activation, differentiation, and functionality of these cells during the active disease (19). In this study, as described for CD4^+^ T cells, the total frequencies and iMFI of CD8^+^ T cells secreting IL-2 remained stable throughout the treatment. Only the total frequencies of the CD8^+^ T secretors cells of TNF-α increased as of D60, and the total frequencies and iMFI of CD8^+^ secretors of IFN-γ, as of D7 and D30. These results suggest the involvement of TNF-α and IFN-γ produced by CD8^+^ T cells in the process of cure.

Like the CD4^+^ T cells, the frequencies of CD8^+^ single secretors of IL-2 were stable, those of TNF-α increased as of D60, and those of IFN-γ increased as of D30. In addition, the frequencies of the double secretors of IL-2 and TNF-α increased as of D60, those of TNF-α and IFN-γ increased as of D60 and D90, and those of double secretors IL-2^+^ and IFN-γ^+^ increased as of D30. Also, the frequencies of the multifunctional CD8^+^ T cells were enhanced after the end of the treatment, at D60. Our results indicate that the participation of cytokine-secreting CD4^+^ T cells starts during the treatment (on day 21), while in contrast, the CD8^+^ response increases as of D60, with the exception of cells producing only IFN-γ or IFN-γ and IL-2. Thus, our results are in agreement with previous investigations that describe exhaustion and reduced functionality of CD8 T cells during disease (19, 52) and increased antigen-specific CD8 T cells in cured patients (21).

Aiming to characterize the evolution of the CD8 T cell functionality during VL, we identified two subpopulations, which had not been described before, in human VL. In this study, CD8^High^ and CD8^Low^ T cells were revealed based on the expression of the CD8 marker on cell membranes. The increase of CD8^High^ T-cell frequencies was correlated with an improvement in the clinical manifestations of VL patients, while CD8^Low^ T cells were mainly present when first diagnosed, which is when patients experienced severe symptoms. We demonstrated that the increase of CD8^High^ was a marker of cure for VL, while the increase of CD8^Low^ was a marker of the disease. In agreement, IFN-γ and/or TNF-α-producing CD8^Low^ T-cell frequencies were also associated with disease in mice infected by *Trypanosoma cruzi* (29, 53) and were reduced after treatment with benznidazole (53). In addition, a predominance of CD8^Low^ T cells was also found in a cohort of children with malaria who were exposed to high parasitic burden, while CD8^High^ T cells were more frequent in children of a low-parasite load area (30). This association of CD8^Low^ with susceptibility to diseases can be explained by the downregulation of the CD8 marker, which is associated with the dysfunction of the Class I Major Histocompatibility Complex (MHC), which in turn compromises the identification of the antigenic and cytotoxic functions and IFN-γ expression (54–56).

Regarding the response to chemotherapy, we showed that MA determined higher total frequencies and iMFI counts of IL-2 and TNF-α-producing CD4 T cells throughout the treatment and up to the cure. In contrast, LAMB had its main impact on the secretion of IL-2, TNF-α, and IFN-γ by CD4^+^ and CD8^+^ T cells, mainly at the beginning on the treatment. More important clinical variables were associated with immunological responses in patients treated with MA than with LAMB. Therefore, LAMB promoted only an earlier immune response, while the immunity in MA-treated patients appeared to be more long-lasting. This difference could be due to the higher toxicity of LAMB to parasites. In fact, LAMB eliminates the parasite load soon after the 5–7 days’ treatment (57–59). Therefore, the stimulus for immune response ends earlier. In contrast, MA treatment does not eliminate all the parasites, which continues stimulating the immune system for longer periods. Actually, LAMB treatment promoted early immune responses of total frequencies of TNF-α-producing T cells that lasted only until day 14 after the beginning of treatment. In contrast, MA was administered until day 21, and higher total frequencies of CD8^+^ T cells were observed until 6 months after the beginning of treatment. There is evidence in literature proving that chemotherapeutic cure is not always sterile. That is why relapses of leishmaniasis can occur in cases of immunosuppression due to HIV co-infection (60) or to treatment of cancer or arthritis (58, 61). Parasites remain in cryptic conditions. In fact, in India, the blood parasite load of VL patients treated with LAMB decreased from 894.07 to 71.72/ml at day 7 after the beginning of therapy (57). Also, in patients with PKDL (post kala-azar dermal leishmaniasis), 8.372 parasites/ml and 194.962 parasites/million nucleated cells were detected by RT-PCR in blood and bone marrow samples, respectively, but no parasites were detected soon after treatment with amphotericin B (58). In contrast, a remaining parasite load was observed in two of six patients following treatment with sodium antimony gluconate (SAG) (58). In agreement, soon after treatment with sodium stibogluconate, negative PCR results were only found in 20% (10/49) of the patients who remained cured until 6 months, while 36% (14/39) of them showed positive PCR and developed PKDL, and an additional 23% (nine) of them had VL relapses including four deaths (59).

However, the detailed analysis of the differentiation of the CD4^+^ and CD8^+^ T-cell cytokine responses revealed that both MA and LAMB promoted immune responses. While MA showed higher influence on IFN-γ secretion, LAMB favored the TNF-α secretion by CD4^+^ lymphocytes. Both drugs, however, generated memory CD4^+^ T-cell responses. In addition, MA treatment increased the frequencies of all types of single- and double-cytokine secretor CD8^+^ T cells, but only LAMB showed higher responses of multifunctional CD8^+^IL-2^+^TNF-α^+^IFN-γ^+^.

Regarding the association of MA treatment with an IFN-γ response, evidence of the involvement of an anti-*Leishmania* specific immune response in the chemotherapeutic success of MA against VL has been previously reported in hamsters (62). The success of MA treatment could therefore be due to both the direct decrease of the parasite load and the direct immunologic Th1 processes induced by the drug (62–64). In our study, the total frequencies, iMFI, and frequencies of CD4^+^ T cells producing only IFN-γ increased until D21, when treatment with MA finished. Furthermore, the CD8^+^IFN-γ-producer phenotype showed also maximal values on D21. In agreement, higher frequencies of IFN-γ positive T cells were described in patients responsive to the SAG (65), and an increased secretion of IFN-γ by splenocytes was disclosed in healthy untreated and in uninfected and Glucantime^®^-treated mice (62). Furthermore, the treatment of patients with SAG promoted the proliferation of CD4 and CD8 T cells but not of B lymphocytes. In addition, it promoted the secretion of IFN-γ and activated both the innate and adaptive immune system by indirectly activating pathways for reactive oxygen species (ROS) and NO generation in VL patients in India (63). Increases of the CD4^+^, CD8^+^, and lymphocyte B-CD19^+^ populations were also reported in *L.* (*L.*) *infantum chagasi*-infected hamsters with VL after treatment with antimonials (62).

On the other hand, and supporting our observation of the influence of LAMB treatment on the rapid enhancement of the TNF-α response, Mondal et al. (66) reported an early immune response involving cytokines and lymphoproliferation after 1 week of VL therapy with a LAMB called Fungisome. In fact, Fungisome promoted a dose-dependent increase of TNF-α secretion 1 week after treatment, which was correlated with cure. In contrast, a negligible elevation of TNF-α was found in the non-curative profile of individuals who underwent relapse within the 6 months of treatment (66). Furthermore, LAMB treatment leads to rapid parasite level decline in BALB/c mouse livers and spleens, associated with a significant enrichment of pathways related to TNF-α (67). Moreover, patients with arthritis who received anti-inflammatory anti-TNF-α treatments with methotrexate (58, 68) or infliximab (61) developed VL, which was further successfully cured with LAMB.

In summary, our data indicate that central-memory CD4^+^TNF-α^+^, CD4^+^IL-2^+^TNF-α^+^IFN-γ^+^ T cells, effector memory CD4^+^TNF-α^+^IFN-γ^+,^ and terminal effector CD4^+^IFN-γ^+^ *Leishmania*-specific T cells are involved in the clinical cure and recovery of the immune response of patients after treatment for VL. In addition, the central-memory CD8^+^IL-2^+^TNF-α^+^IFN-γ^+^, the effector memory CD8^+^TNF-α^+^IFN-γ^+^, and terminal effector CD8^+^IFN-γ^+^ T cells, together with CD8^+^TNF-α^+^, CD8^+^IL-2^+^TNF-α^+^, and CD8^+^IL-2^+^IFN-γ^+^ T cells, are also relevant in the healing process. Noteworthy, the frequencies of the central-memory CD4^+^ and CD8^+^IL-2^+^TNF-α^+^IFN-γ^+^ T cells, which ensure the memory response against parasite reinfection, are significantly enhanced in the cured patients, starting as of D60. In this study, we also described that the subset of the non-functional CD8^Low^ population (54) is predominant in VL untreated patients and decreases along chemotherapy, as reported before for malaria (30) and Chagas disease (29, 53) patients. Conversely, a progressive increase of the CD8^High^ subset was observed along the treatment, and these cells reached normal levels as of D21 and until the complete cure. Both treatments with LAMB and MA were efficacious, and all patients achieved an effective clinical cure with no relapse for at least 2 years. To our knowledge, this is the first description of the evolution and differentiation of the CD4 and CD8 T-cell immune responses in patients treated for VL.

## Data Availability Statement

The original contributions presented in the study are included in the article/**Supplementary Materials**. Further inquiries can be directed to the corresponding author.

## Ethics Statement

The studies involving human participants were reviewed and approved by the Research and Ethics Committee of the Federal University of Sergipe (UFS)-University Hospital, Aracaju, Sergipe State (SE), Brazil (CAAE 0162.0.107.000-09). The experiments were performed according to the ethical standards of the Declaration of Helsinki and followed the guidelines and regulations of the Brazilian National Council of Health resolution 196/96. The patients/participants provided their written informed consent to participate in this study.

## Author Contributions

LR, AB, LB, MC, NF, GC, and LM conducted the experiments. LR, AB, LB, and MC acquired the data. LR, CP-d-S, and CC analyzed the data. AJ, CC, and RA designed the research studies. LR, CP-d-S, and CC wrote the manuscript. All authors contributed to the article and approved the submitted version.

## Funding

This work was supported by Conselho Nacional de Desenvolvimento Científico e Tecnológico (CNPQ) fellowship and grant Proc. 309776/2018-0, and CNPq 429246/2016-1 to RA; CNPQ fellowship and grant Proc. 307741/2017-6 to AJ; and CNPQ fellowship and grant Proc. 304764/2018-3 and Fundação Carlos Chagas de Amparo à Pesquisa do Estado de Rio de Janeiro (FAPERJ) fellowships and grants E-26/202.903/2017 and E-26/200.887/2021, and grant E-26/010002419/2019 to CP-d-S. LR was a recipient of an MSc fellowship from CNPQ Proc. 130572/2018-7 and of a PhD fellowship from Coordenação de Aperfeiçoamento de Pessoal de Nível Superior (CAPES) Proc. 88887.485785/2020-00, Brazil.

## Conflict of Interest

The authors declare that the research was conducted in the absence of any commercial or financial relationships that could be construed as a potential conflict of interest.

## Publisher’s Note

All claims expressed in this article are solely those of the authors and do not necessarily represent those of their affiliated organizations, or those of the publisher, the editors and the reviewers. Any product that may be evaluated in this article, or claim that may be made by its manufacturer, is not guaranteed or endorsed by the publisher.

## Supplementary Material

Supplementary Figure 1 Gating strategy used to analyze Multifunctional T cells populations using multiparameter flow cytometry. Lymphocytes were defined by using FSC-A versus SSC-A parameters, after we selected CD4 and CD8 populations included in the CD3 positive population. Then we excluded naïve T cells population (CCR7^+^/CD45RA^+^) and we analyzed the cytokine (IL-2, TNF-α, and IFN- γ) expression from CD4 and CD8 T cells. We performed Boolean gate strategy to generate combinations between cytokine expression by T cells and we observed different kind of phenotypes as single, double or triple cytokine expression. These data were analyzed using FlowJo^®^ software.

Supplementary Figure 2 Evolution of the relative proportions of each CD4^+^ cytokine-expressing subtype along the time of treatment. The graph represents the variation of the percent of each type of T cell among all cytokine-expressing CD4^+^ T cells along the time.

Supplementary Figure 3 Evolution of the relative proportions of each CD8^+^ cytokine-expressing subtype along the time of treatment. The graph represents the variation of the percent of each type of T cell among all cytokine-expressing CD8^+^ T cells along the time.

Supplementary Figure 4 Evolution of the relative proportions of each CD4^+^ cytokine-expressing subtype considering separately the patients treated with LAMB or MA. The graph represents the variation of the percent of each type of T cell among all cytokine-expressing CD4^+^ T cells along the time.

Supplementary Figure 5 Evolution of the relative proportions of each CD8^+^ cytokine-expressing subtype considering separately the patients treated with LAMB or MA. The graph represents the variation of the percent of each type of T cell among all cytokine-expressing CD8^+^ T cells along the time.

## References

[1] Alvar J Vélez ID Bern C Herrero M Desjeux P Cano J. Leishmaniasis Worldwide and Global Estimates of Its Incidence. PLoS One (2012) 7(5):e35671. doi: 10.1371/journal.pone.0035671 22693548 PMC3365071

[2] World Health Organization. Leishmaniasis (2019). Available at: https://www.who.int/news-room/fact-sheets/detail/leishmaniasis (Accessed Last Access August 18th, 2021).

[3] Organização Pan-Americana de Saúde Organização Mundial de Saúde. Leishmanioses - Informe Epidemiológico Das Américas. Available at: https://iris.paho.org/bitstream/handle/10665.2/50505/2019-cde-leish-informe-epi-das-americas.pdf?ua=1 (Accessed Last access August 18th, 2021).

[4] Salomão De Azevedo T Lorenz C Chiaravalloti-Neto F. Risk Mapping of Visceral Leishmaniasis in Brazil. J Braz Soc Trop Med (2019) 52:1–5. doi: 10.1590/0037-8682-0240-2019 31778399

[5] Araújo-Santos T Andrade BB Gil-Santana L Luz NF Dos Santos PL De Oliveira FA. Anti-Parasite Therapy Drives Changes in Human Visceral Leishmaniasis-Associated Inflammatory Balance. Sci Rep (2017) 7:1–8. doi: 10.1038/s41598-017-04595-8 28659627 PMC5489532

[6] Ghosh MK Ghosh AK Addy M Nandy A Ghose AC. Subpopulations of T Lymphocytes in the Peripheral Blood and Lymph Nodes of Indian Kala-Azar Patients. Med Microbiol Immunol (1996) 185:183–7. doi: 10.1007/s004300050029 9007824

[7] Rohtagi A Agarwall SK Mridula Bose D Chattopadhya D Saha K. Blood, Bone Marrow and Splenic Lymphocyte Subset Profiles in Indian Visceral Leishmaniasis. Trans R Soc Trop Med Hyg (1996) 90:431–4. doi: 10.1016/s0035-9203(96)90537-4 8882198

[8] Hailu A Van Baarle D Knol GJ Berhe N Miedema F Kager PA. T Cell Subset and Cytokine Profiles in Human Visceral Leishmaniasis During Active and Asymptomatic or Sub-Clinical Infection With Leishmania Donovani. Clin Immunol (2005) 117:182–91. doi: 10.1016/j.clim.2005.06.015 16125466

[9] Kuschnir RC Pereira LS Dutra MRT de Paula L Silva-Freitas ML Corrêa-Castro G. High Levels of Anti-Leishmania IgG3 and Low CD4+ T Cells Count Were Associated With Relapses in Visceral Leishmaniasis. BMC Infect Dis (2021) 21:1–14. doi: 10.1186/s12879-021-06051-5 33874901 PMC8056614

[10] Carvalho EM Bacellar O Barral A Badaro R Johnson WD. Antigen-Specific Immunosuppression in Visceral Leishmaniasis Is Cell Mediated. J Clin Invest (1989) 83:860–4. doi: 10.1172/JCI113969 PMC303759 2522103

[11] Santos MLB Nico D de Oliveira FA Barreto AS Palatnik-de-Sousa I Carrillo E. Leishmania Donovani Nucleoside Hydrolase (NH36) Domains Induce T-Cell Cytokine Responses in Human Visceral Leishmaniasis. Front Immunol (2017) 8:227. doi: 10.3389/fimmu.2017.00227 28321221 PMC5338038

[12] Dayakar A Chandrasekaran S Kuchipudi SV Kalangi SK. Cytokines: Key Determinants of Resistance or Disease Progression in Visceral Leishmaniasis: Opportunities for Novel Diagnostics and Immunotherapy. Front Immunol (2019) 10:670. doi: 10.3389/fimmu.2019.00670 31024534 PMC6459942

[13] Kumar P Misra P Thakur CP Saurabh A Rishi N Mitra DK. T Cell Suppression in the Bone Marrow of Visceral Leishmaniasis Patients: Impact of Parasite Load. Clin Exp Immunol (2018) 191:318–27. doi: 10.1111/cei.13074 PMC5801524 29058314

[14] Rai AK Thakur CP Singh A Seth T Srivastava SK Singh P. Regulatory T Cells Suppress T Cell Activation at the Pathologic Site of Human Visceral Leishmaniasis. PLoS One (2012) 7:1–11. doi: 10.1371/journal.pone.0031551 PMC3275558 22347492

[15] Rodrigues-Neto JF Monteiro GR Keesen TSL Lacerda HG Carvalho EM Jeronimo SMB. CD45RO+ T Cells and T Cell Activation in the Long-Lasting Immunity After Leishmania Infantum Infection. Am J Trop Med Hyg (2018) 98:875–82. doi: 10.4269/ajtmh.16-0747 PMC5930877 29280433

[16] Stäger S Rafati S. CD8+ T Cells in Leishmania Infections: Friends or Foes? Front Immunol (2012) 3:5. doi: 10.3389/fimmu.2012.00005 22566891 PMC3342007

[17] Mary C Auriault V Faugère B Dessein AJ. Control of Leishmania Infantum Infection Is Associated With CD8+ and Gamma Interferon- and Interleukin-5-Producing CD4+ Antigen-Specific T Cells. Infect Immun (1999) 67:5559–66. doi: 10.1128/iai.67.11.5559-5566.1999 PMC96926 10531200

[18] Clarêncio J de Oliveira CI Favali C Medina O Caldas A Henrique Costa C. Could the Lower Frequency of CD81CD181CD45RO1 Lymphocytes be Biomarkers of Human VL? Int Immunol (2009) 21:137–44. doi: 10.1093/intimm/dxn131 19088063

[19] Singh B Bhushan Chauhan S Kumar R Singh SS Ng S Amante F. A Molecular Signature for CD8+ T Cells From Visceral Leishmaniasis Patients. Parasite Immunol (2019) 41:1–8. doi: 10.1111/pim.12669 31494954

[20] Jawed JJ Dutta S Majumdar S. Functional Aspects of T Cell Diversity in Visceral Leishmaniasis. BioMed Pharmacother (2019) 117:1–4. doi: 10.1016/j.biopha.2019.109098 31195352

[21] Kaushal H Bras-Gonçalves R Negi NS Lemesre JL Papierok G Salotra P. Role of CD8+ T Cells in Protection Against Leishmania Donovani Infection in Healed Visceral Leishmaniasis Individuals. BMC Infect Dis (2014) 14:1–7. doi: 10.1186/s12879-014-0653-6 25471494 PMC4258298

[22] Carrillo E Fernandez L Ibarra-Meneses AV Santos MLB Nico D de Luca PM. F1 Domain of the Leishmania (Leishmania) Donovani Nucleoside Hydrolase Promotes a Th1 Response in Leishmania (Leishmania) Infantum Cured Patients and in Asymptomatic Individuals Living in an Endemic Area of Leishmaniasis. Front Immunol (2017) 8:750. doi: 10.3389/fimmu.2017.00750 28747911 PMC5506215

[23] Das A Ali N. Vaccine Prospects of Killed But Metabolically Active Leishmania Against Visceral Leishmaniasis. Expert Rev Vaccines (2012) 11:783–5. doi: 10.1586/erv.12.50 22913255

[24] Kubar J Fragaki K. Recombinant DNA-Derived Leishmania Proteins: From the Laboratory to the Field. Lancet Infect Dis (2005) 5:107–14. doi: 10.1016/s1473-3099(05)01282-x 15680780

[25] De Luca PM Macedo ABB. Cutaneous Leishmaniasis Vaccination: A Matter of Quality. Front Immunol (2016) 7:151. doi: 10.3389/fimmu.2016.00151 27148270 PMC4838622

[26] Seder RA Darrah PA Roederer M. T-Cell Quality in Memory and Protection: Implications for Vaccine Design. Nat Rev Immunol (2008) 8:247–58. doi: 10.1038/nri2274 18323851

[27] Darrah PA Patel DT De Luca PM Lindsay RWB Davey DF Flynn BJ. Multifunctional TH1 Cells Define a Correlate of Vaccine-Mediated Protection Against Leishmania Major. Nat Med (2007) 13:843–50. doi: 10.1007/s10959-016-0701-9 17558415

[28] Ouyang L Li X Liang Z Yang D Gong F Shen G. CD8low T-Cell Subpopulation Is Increased in Patients With Chronic Hepatitis B Virus Infection. Mol Immunol (2013) 56:698–704. doi: 10.1016/j.molimm.2013.07.003 23933510

[29] Grisotto MG D’Império Lima MR Marinho CRF Tadokoro CE Abrahamsohn IA Alvarez JM. Most Parasite-Specific CD8+ Cells in Trypanosoma Cruzi-Infected Chronic Mice Are Down-Regulated for T-Cell Receptor-αβ and CD8 Molecules. Immunology (2001) 102:209–17. doi: 10.1046/j.1365-2567.2001.01170.x PMC1783160 11260326

[30] Falanga YT Frascoli M Kaymaz Y Forconi C Ong’echa JM Bailey JA. High Pathogen Burden in Childhood Promotes the Development of Unconventional Innate-Like CD8+ T Cells. JCI Insight (2017) 2:1–17. doi: 10.1172/jci.insight.93814 PMC5543908 28768916

[31] Ding Z-D Zheng J-F Song C-B Fu Y-J Xu J-J Jiang Y-J. Decreased CD4+CD8low T Cells in Early HIV Infection Are Associated With Rapid Disease Progression. Cytokine (2019) 125:1–7. doi: 10.1016/j.cyto.2019.154801 31442680

[32] Romero GAS Costa DL Costa CHN de Almeida RP de Melo EV de Carvalho SFG. Efficacy and Safety of Available Treatments for Visceral Leishmaniasis in Brazil: A Multicenter, Randomized, Open Label Trial. PLoS Negl Trop Dis (2017) 11:1–25. doi: 10.1371/journal.pntd.0005706 PMC5507560 28662034

[33] Ministério da Saúde. Guia De Vigilância Em Saúde. 3a Edição., Ed. Ministério Da Saúde, Secretaria De Vigilância Em Saúde, Coordenação-Geral De Desenvolvimento Da Epidemiologia Em Serviços, Secretaria De Vigilância Em Saúde Brasília: Ministério Da Saúde. Brazilian Ministry of Health: Brasília, DF. (2019). pp. 502–22.

[34] Sundar S Jha TK Thakur CP Mishra M Singh VP Buffels R. Single-Dose Liposomal Amphotericin B in the Treatment of Visceral Leishmaniasis in India: A Multicenter Study. Clin Infect Dis (2003) 37:800–4. doi: 10.1086/377542 12955641

[35] Rodrigues V Cordeiro-Da-Silva A Laforge M Silvestre R Estaquier J. Regulation of Immunity During Visceral Leishmania Infection. Parasites Vectors (2016) 9:1–13. doi: 10.1186/s13071-016-1412-x 26932389 PMC4774109

[36] Santos PL de Oliveira FA Santos MLB Cunha LCS Lino MTB de Oliveira MFS. The Severity of Visceral Leishmaniasis Correlates With Elevated Levels of Serum IL-6, IL-27 and sCD14. PLoS Negl Trop Dis (2016) 10:1–16. doi: 10.1371/journal.pntd.0004375 PMC4729473 26814478

[37] Goto H Prianti MDG. Immunoactivation and Immunopathogeny During Active Visceral Leishmaniasis. Rev Inst Med Trop Sao Paulo (2009) 51:241–6. doi: 10.1590/S0036-46652009000500002 19893975

[38] Kaye PM Svensson M Ato M Maroof A Polley R Stager S. The Immunopathology of Experimental Visceral Leishmaniasis. Immunol Rev (2004) 201:239–53. doi: 10.1111/j.0105-2896.2004.00188.x 15361245

[39] Peruhype-Magalhães V Martins-Filho OA Prata A Silva LDA Rabello A Teixeira-Carvalho A. Immune Response in Human Visceral Leishmaniasis: Analysis of the Correlation Between Innate Immunity Cytokine Profile and Disease Outcome. Scand J Immunol (2005) 62:487–95. doi: 10.1111/j.1365-3083.2005.01686.x 16305646

[40] Singh N Sundar S. Combined Neutralization of Interferon Gamma and Tumor Necrosis Factor Alpha Induces IL-4 Production But has No Direct Additive Impact on Parasite Burden in Splenic Cultures of Human Visceral Leishmaniasis. PLoS One (2018) 13:1–11. doi: 10.1371/journal.pone.0199817 PMC6023118 29953494

[41] Kumar R Singh N Gautam S Singh OP Gidwani K Rai M. Leishmania Specific CD4 T Cells Release Ifnγ That Limits Parasite Replication in Patients With Visceral Leishmaniasis. PLoS Negl Trop Dis (2014) 8(10):e3198. doi: 10.1371/journal.pntd.0003198 25275531 PMC4183461

[42] Samant M Sahu U Pandey SC Khare P. Role of Cytokines in Experimental and Human Visceral Leishmaniasis. Front Cell Infect Microbiol (2021) 11:624009. doi: 10.3389/fcimb.2021.624009 33680991 PMC7930837

[43] Costa DL Rocha RL Carvalho RMA Lima-Neto AS Harhay MO Costa CHN. Serum Cytokines Associated With Severity and Complications of Kala-Azar. Pathog Glob Health (2013) 107:78–87. doi: 10.1179/2047773213Y.0000000078 23683334 PMC4001482

[44] Ho JL Badaró R Schwartz A Dinarello CA Gelfand JA Sobel J. Diminished *In Vitro* Production of Interleukin-1 and Tumor Necrosis Factor-α During Acute Visceral Leishmaniasis and Recovery After Therapy. J Infect Dis (1992) 165:1094–102. doi: 10.1093/infdis/165.6.1094 1583328

[45] Yadav S Prakash J Singh OP Gedda MR Chauhan SB Sundar S. IFN-γ+ CD4+T Cell-Driven Prophylactic Potential of Recombinant LDBPK_252400 Hypothetical Protein of Leishmania Donovani Against Visceral Leishmaniasis. Cell Immunol (2021) 361:104272. doi: 10.1016/j.cellimm.2020.104272 33445051 PMC7890570

[46] Macedo ABB Sánchez-Arcila JC Schubach AO Mendonça SCF Marins-Dos-Santos A De Fatima Madeira M. Multifunctional CD4+T Cells in Patients With American Cutaneous Leishmaniasis. Clin Exp Immunol (2012) 167:505–13. doi: 10.1111/j.1365-2249.2011.04536.x PMC3374283 22288594

[47] Lakhal-Naouar I Slike BM Aronson NE Marovich MA. The Immunology of a Healing Response in Cutaneous Leishmaniasis Treated With Localized Heat or Systemic Antimonial Therapy. PLoS Negl Trop Dis (2015) 9:1–17. doi: 10.1371/journal.pntd.0004178 PMC4618688 26485398

[48] Zaph C Uzonna J Beverley SM Scott P. Central Memory T Cells Mediate Long-Term Immunity to Leishmania Major in the Absence of Persistent Parasites. Nat Med (2004) 10:1104–10. doi: 10.1038/nm1108 15448686

[49] Goto Y Bhatia A Raman VS Liang H Mohamath R Picone AF. KSAC, the First Defined Polyprotein Vaccine Candidate for Visceral Leishmaniasis. Clin Vaccine Immunol (2011) 18:1118–24. doi: 10.1128/CVI.05024-11 PMC3147330 21632891

[50] Nico D Gomes DC Palatnik-de-Sousa I Morrot A Palatnik M Palatnik-de-Sousa CB. Leishmania Donovani Nucleoside Hydrolase Terminal Domains in Cross-Protective Immunotherapy Against Leishmania Amazonensis Murine Infection. Front Immunol (2014) 5:273. doi: 10.3389/fimmu.2014.00273 24966857 PMC4052736

[51] Sabur A Bhowmick S Chhajer R Ejazi SA Didwania N Asad M. Liposomal Elongation Factor-1α Triggers Effector CD4 and CD8 T Cells for Induction of Long-Lasting Protective Immunity Against Visceral Leishmaniasis. Front Immunol (2018) 9:18. doi: 10.3389/fimmu.2018.00018 29441060 PMC5797590

[52] Gautam S Kumar R Singh N Singh AK Rai M Sacks D. CD8 T Cell Exhaustion in Human Visceral Leishmaniasis. J Infect Dis (2014) 209:290–9. doi: 10.1093/infdis/jit401 PMC3873784 23922369

[53] Marins-dos-Santos A Olivieri BP Ferreira-Reis R de Meis J Silva AA de Araújo-Jorge TC. Cd8low T Cells Expanded Following Acute Trypanosoma Cruzi Infection and Benznidazole Treatment Are a Relevant Subset of Ifn-γ Producers. PLoS Negl Trop Dis (2020) 14:1–15. doi: 10.1371/JOURNAL.PNTD.0008969 PMC7785226 33347472

[54] Xiao Z Mescher M Jameson S. Detuning CD8 T Cells: Down-Regulation of CD8 Expression, Tetramer Binding, and Response During CTL Activation. J Exp Med (2007) 204:2667–77. doi: 10.1084/JEM.20062376 PMC2118473 17954566

[55] Kienzle N Baz A Kelso A. Profiling the CD8low Phenotype, an Alternative Career Choice for CD8 T Cells During Primary Differentiation. Immunol Cell Biol (2004) 82:75–83. doi: 10.1111/j.1440-1711.2004.01210.x 14984598

[56] Kienzle N Buttigieg K Groves P Kawula T Kelso A. A Clonal Culture System Demonstrates That IL-4 Induces a Subpopulation of Noncytolytic T Cells With Low CD8, Perforin, and Granzyme Expression 1. J Immunol (2002) 168:1672–81. doi: 10.4049/jimmunol.168.4.1672 11823496

[57] Sudarshan M Weirather JL Wilson ME Sundar S. Study of Parasite Kinetics With Antileishmanial Drugs Using Real-Time Quantitative PCR in Indian Visceral Leishmaniasis. J Antimicrob Chemother (2011) 66:1751–5. doi: 10.1093/jac/dkr185 PMC3133483 21609983

[58] Verma S Kumar R Katara GK Singh LC Negi NS. Quantification of Parasite Load in Clinical Samples of Leishmaniasis Patients: IL-10 Level Correlates With Parasite Load in Visceral Leishmaniasis. PLoS One (2010) 5:1–8. doi: 10.1371/journal.pone.0010107 PMC2852412 20404924

[59] Osman OF Oskam L Zijlstra EE El-Hassan AM El-Naeim DA Kager PA. Use of the Polymerase Chain Reaction to Assess the Success of Visceral Leishmaniasis Treatment. Trans R Soc Trop Med Hyg (1998) 92:397–400. doi: 10.1016/S0035-9203(98)91063-X 9850390

[60] Sundar S Singh A. Chemotherapeutics of Visceral Leishmaniasis: Present and Future Developments. Parasitology (2018) 145:481–9. doi: 10.1017/S0031182017002116 PMC5984184 29215329

[61] Bousquet E Mura F Villain M Rivière S Konate A Schneider C. Complications Infectieuses Liées Au Traitement Par Anti-TNF: À Propos De Deux Cas De Leishmaniose Infectious Complications in Patients Treated With Anti-TNF-Alpha: Two Cases of Leishmaniasis. J Fr Ophtalmol (2012) 35:695–9. doi: 10.1016/j.jfo.2012.06.007 22925844

[62] Freitas EO Nico D Alves-Silva MV Morrot A Clinch K Evans GB. Immucillins ImmA and ImmH Are Effective and Non-Toxic in the Treatment of Experimental Visceral Leishmaniasis. PLoS Negl Trop Dis (2015) 9(12):e0004297. doi: 10.1371/JOURNAL.PNTD.0004297 26701750 PMC4689457

[63] Haldar AK Sen P Roy S. Use of Antimony in the Treatment of Leishmaniasis: Current Status and Future Directions. Mol Biol Int (2011) 2011:1–23. doi: 10.4061/2011/571242 PMC3196053 22091408

[64] Roychoudhury J Ali N. Sodium Stibogluconate: Therapeutic Use in the Management of Leishmaniasis. Indian J Geo Marine Sci (2008) 45:16–22.

[65] Thakur CP Mitra DK Narayan S. Skewing of Cytokine Profiles Towards T Helper Cell Type 2 Response in Visceral Leishmaniasis Patients Unresponsive to Sodium Antimony Gluconate. Trans R Soc Trop Med Hyg (2003) 97:409–12. doi: 10.1016/S0035-9203(03)90071-X 15259468

[66] Mondal S Bhattacharya P Rahaman M Ali N Goswami RP. A Curative Immune Profile One Week After Treatment of Indian Kala-Azar Patients Predicts Success With a Short-Course Liposomal Amphotericin B Therapy. PLoS Negl Trop Dis (2010) 4:1–8. doi: 10.1371/journal.pntd.0000764 PMC2910702 20668544

[67] Forrester S Siefert K Ashwin H Brown N Zelmar A James S. Tissue-Specific Transcriptomic Changes Associated With AmBisome^®^ Treatment of BALB/c Mice With Experimental Visceral Leishmaniasis. Wellcome Open Res (2019) 4:1–21. doi: 10.12688/wellcomeopenres.15606.1 31976381 PMC6961418

[68] Venizelos I Tatsiou Z Papathomas TG Orazi A. Visceral Leishmaniasis in a Rheumatoid Arthritis Patient Treated With Methotrexate. Int J Infect Dis (2009) 13:169–72. doi: 10.1016/j.ijid.2008.09.012 19026580

