# Supplementary figures and images for: Multifunctional, TNF-α and IFN-γ-Secreting CD4 and CD8 T Cells and CD8High T Cells Are Associated With the Cure of Human Visceral Leishmaniasis
Source: Front Immunol. 2021 Oct 28;12:773983. doi: 10.3389/fimmu.2021.773983 (PMC8581227; doi:10.3389/fimmu.2021.773983)

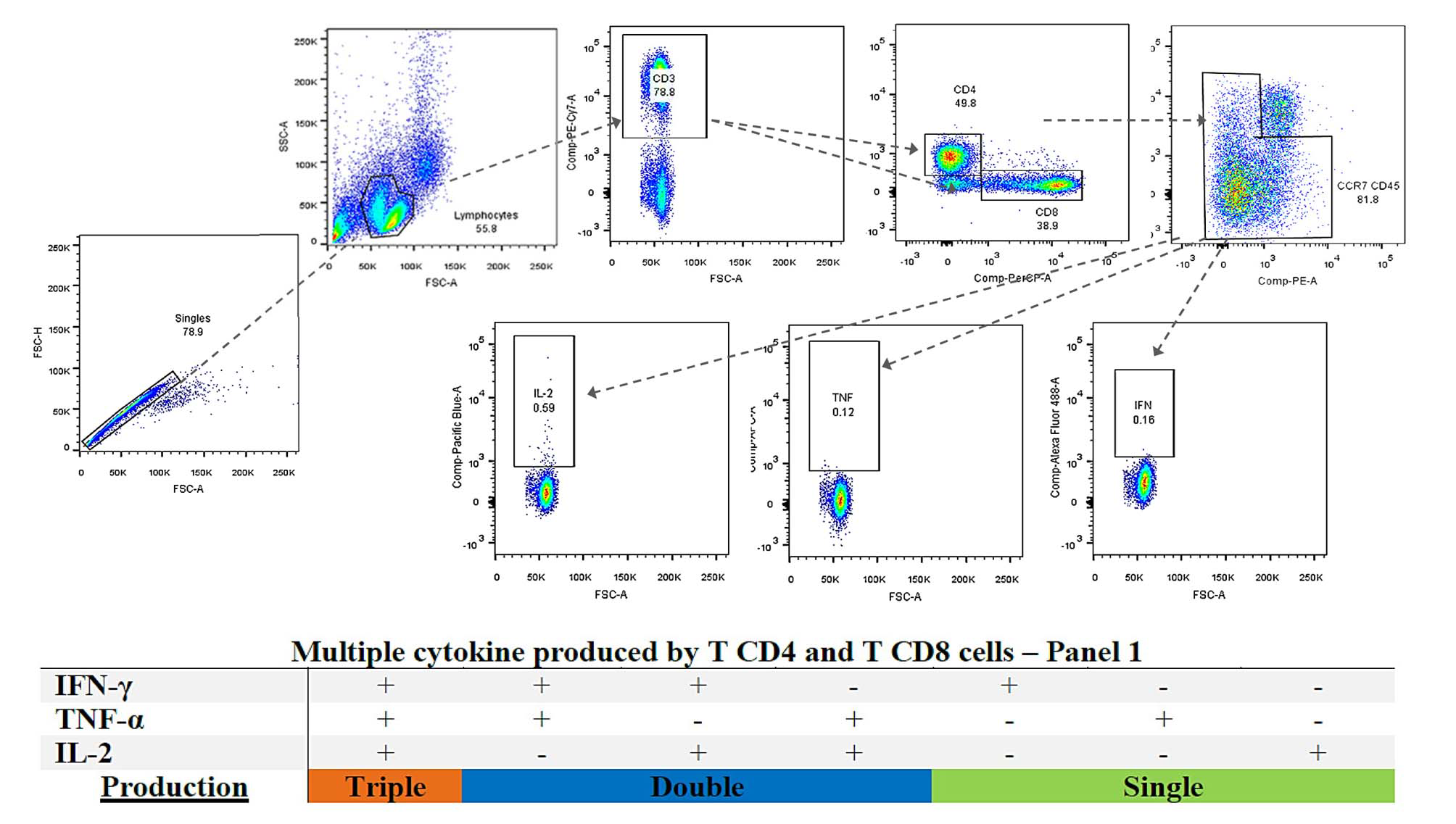

Supplement: Supplementary Figure 1 — Gating strategy used to analyze Multifunctional T cells populations using multiparameter flow cytometry. Lymphocytes were defined by using FSC-A versus SSC-A parameters, after we selected CD4 and CD8 populations included in the CD3 positive population. Then we excluded naïve T cells population (CCR7+/CD45RA+) and we analyzed the cytokine (IL-2, TNF-α, and IFN- γ) expression from CD4 and CD8 T cells. We performed Boolean gate strategy to generate combinations between cytokine expression by T cells and we observed different kind of phenotypes as single, double or triple cytokine expression. These data were analyzed using FlowJo® software. [file Image_1.tif]

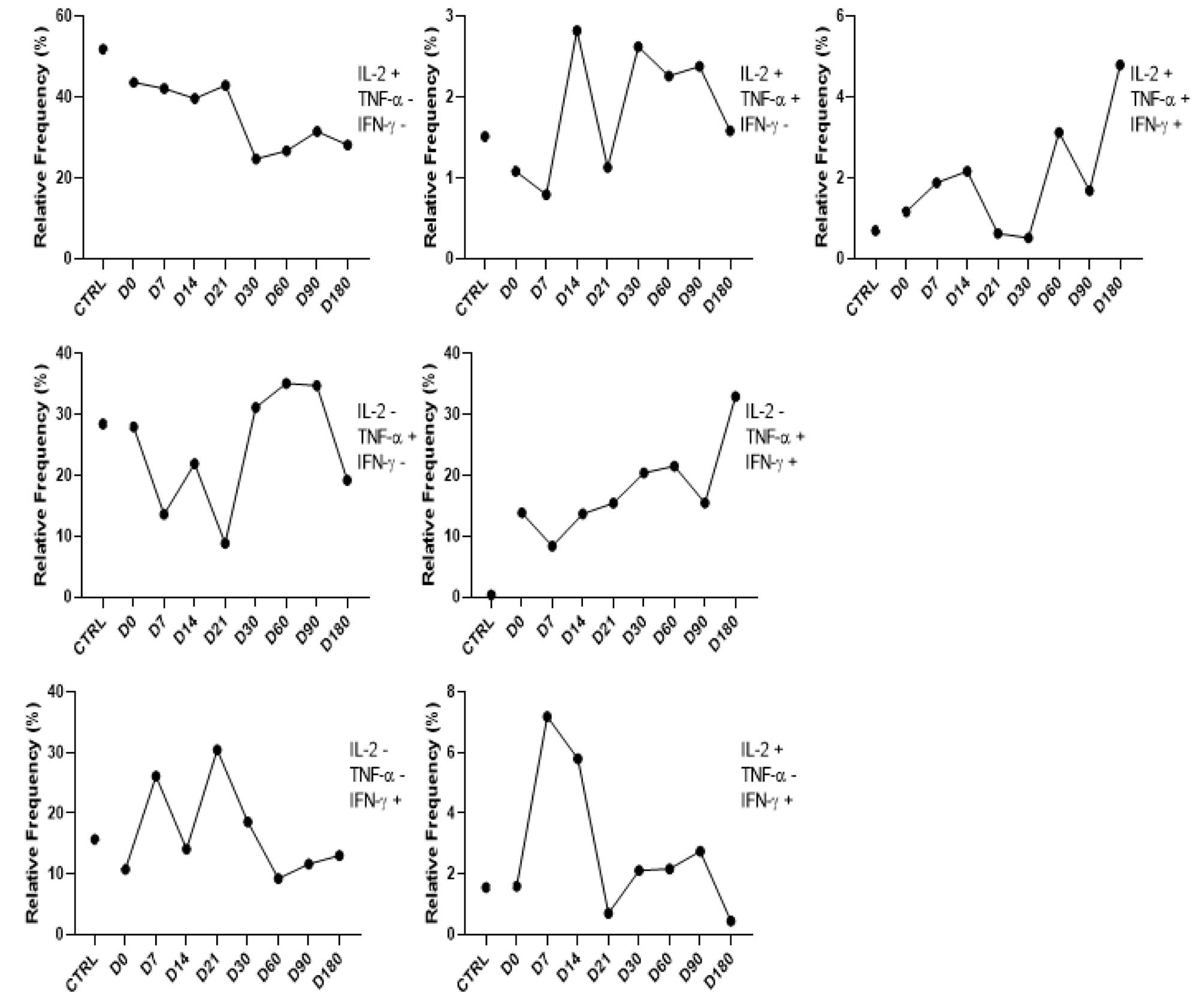

Supplement: Supplementary Figure 2 — Evolution of the relative proportions of each CD4+ cytokine-expressing subtype along the time of treatment. The graph represents the variation of the percent of each type of T cell among all cytokine-expressing CD4+ T cells along the time. [file Image_2.tif]

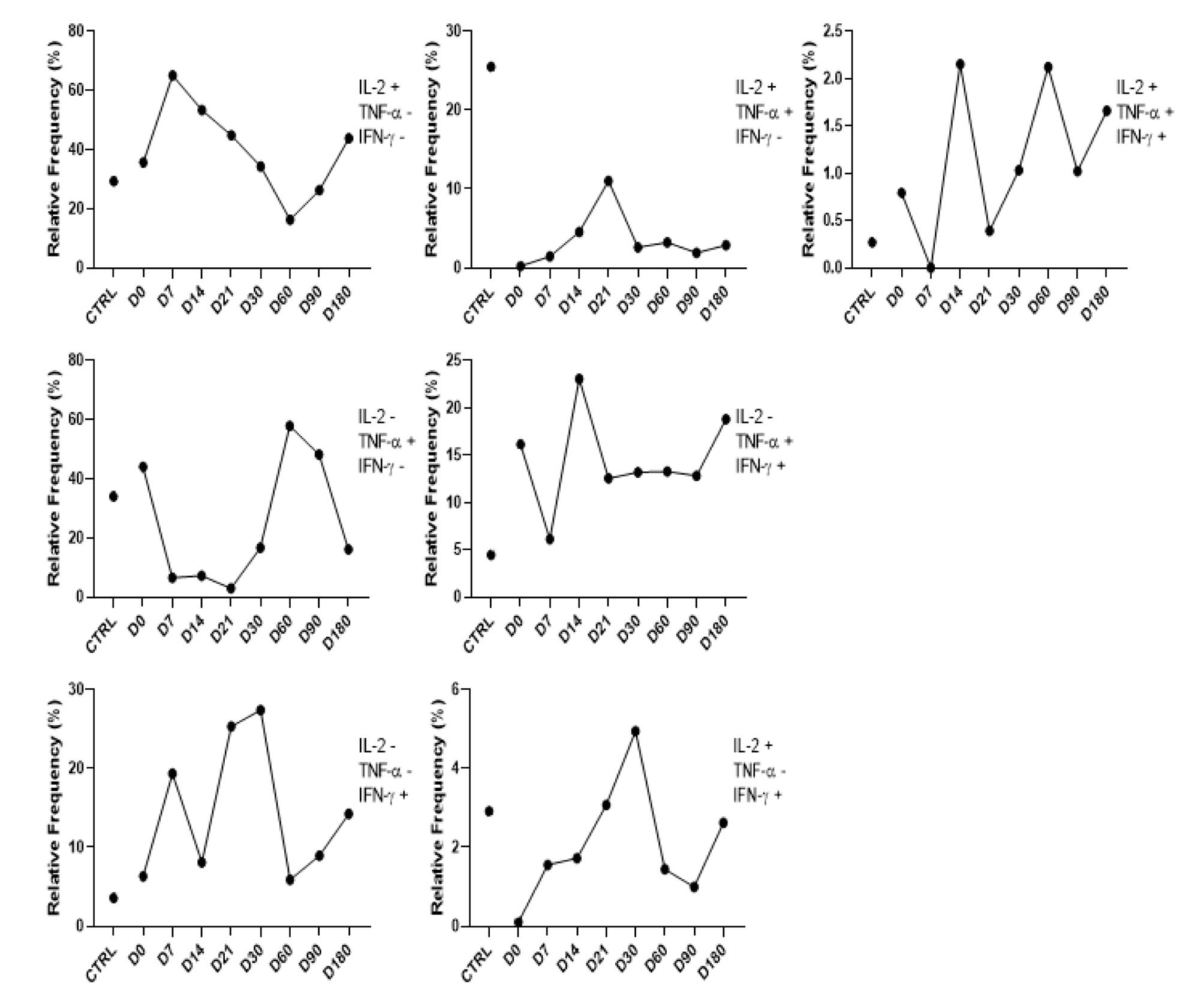

Supplement: Supplementary Figure 3 — Evolution of the relative proportions of each CD8+ cytokine-expressing subtype along the time of treatment. The graph represents the variation of the percent of each type of T cell among all cytokine-expressing CD8+ T cells along the time. [file Image_3.tif]

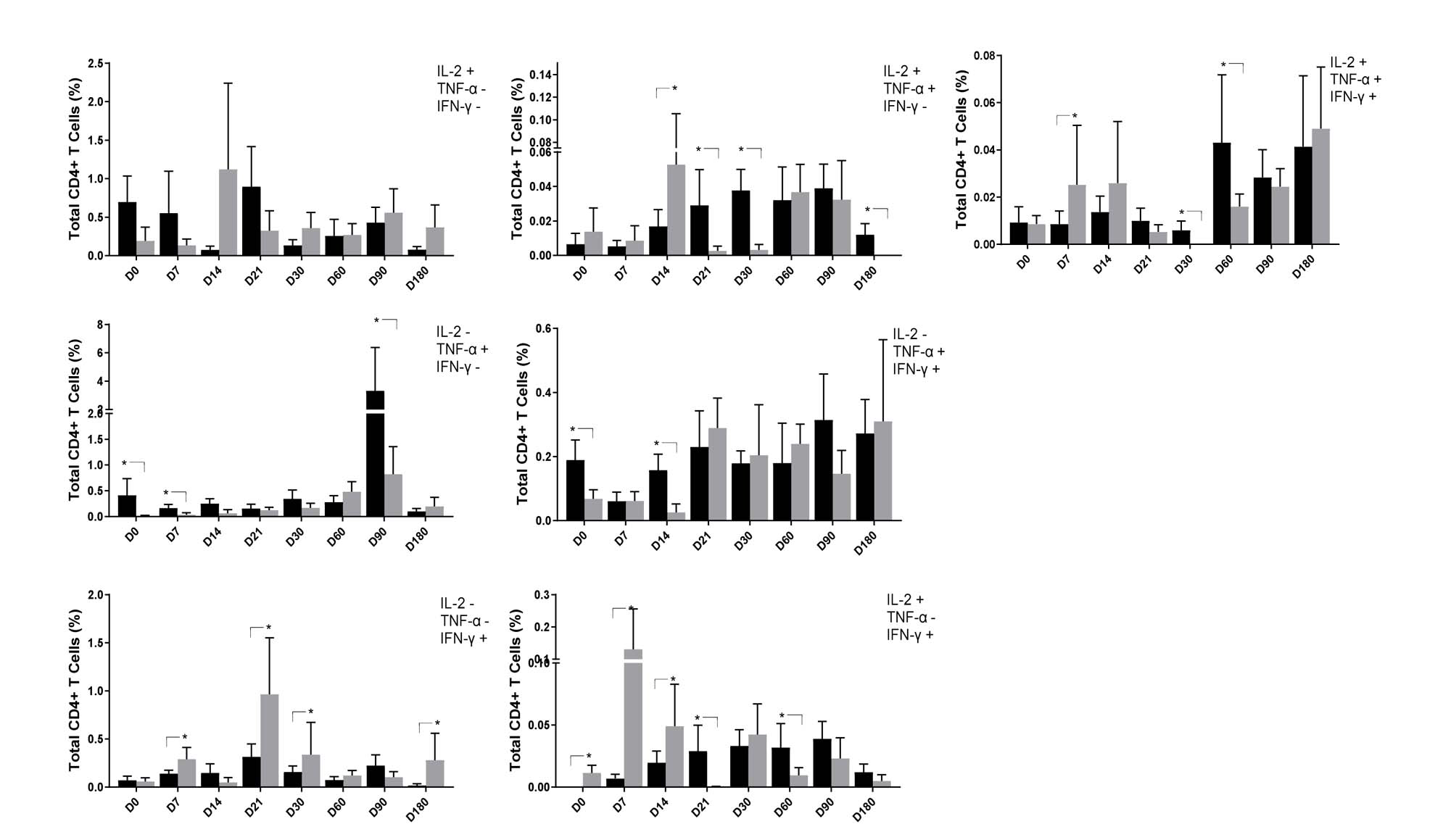

Supplement: Supplementary Figure 4 — Evolution of the relative proportions of each CD4+ cytokine-expressing subtype considering separately the patients treated with LAMB or MA. The graph represents the variation of the percent of each type of T cell among all cytokine-expressing CD4+ T cells along the time. [file Image_4.tif]

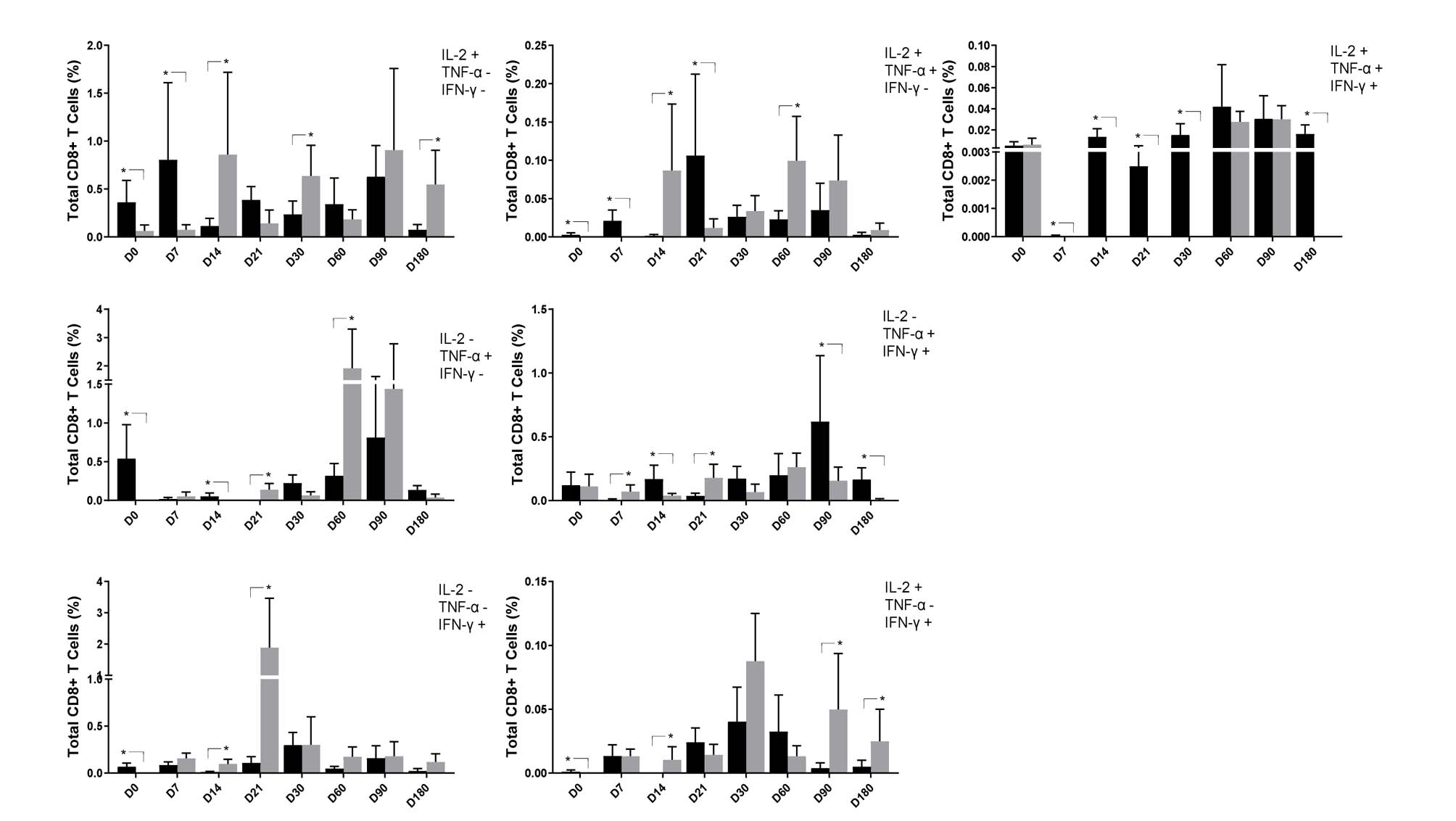

Supplement: Supplementary Figure 5 — Evolution of the relative proportions of each CD8+ cytokine-expressing subtype considering separately the patients treated with LAMB or MA. The graph represents the variation of the percent of each type of T cell among all cytokine-expressing CD8+ T cells along the time. [file Image_5.tif]
